# Blocking IL-6 signaling prevents astrocyte-induced neurodegeneration in an iPSC-based model of Parkinson’s disease

**DOI:** 10.1172/jci.insight.163359

**Published:** 2024-02-08

**Authors:** Meritxell Pons-Espinal, Lucas Blasco-Agell, Irene Fernandez-Carasa, Pol Andrés-Benito, Angelique di Domenico, Yvonne Richaud-Patin, Valentina Baruffi, Laura Marruecos, Lluís Espinosa, Alicia Garrido, Eduardo Tolosa, Michael J. Edel, Manel Juan Otero, José Luis Mosquera, Isidre Ferrer, Angel Raya, Antonella Consiglio

**Affiliations:** 1 Department of Pathology and Experimental Therapeutics, Bellvitge University Hospital-IDIBELL, Hospitalet de Llobregat, Spain.; 2 Institute of Biomedicine (IBUB) of the University of Barcelona (UB), Barcelona, Spain.; 3 Neuropathology, Bellvitge Biomedical Research Institute (IDIBELL), Hospital Duran i Reynals, Hospitalet de Llobregat, Spain.; 4 Network Research Center of Neurodegenerative Diseases (CIBERNED), Hospitalet de Llobregat, Spain.; 5 Institute for Neurodegenerative Diseases, University California San Francisco, San Francisco, California, USA.; 6 Regenerative Medicine Program, IDIBELL, and Program for Clinical Translation of Regenerative Medicine in Catalonia (P-CMRC), Hospital Duran i Reynals, Hospitalet de Llobregat, Barcelona, Spain.; 7 Centre for Networked Biomedical Research on Bioengineering, Biomaterials and Nanomedicine (CIBER-BBN), Madrid, Spain.; 8 Hospital del Mar Research Institute, CIBERONC, Barcelona, Spain.; 9 Centre for Networked Biomedical Research on Neurodegenerative Diseases (CIBERNED), Madrid, Spain.; 10 Department of Neurology, Hospital Clínic de Barcelona, Institut d’Investigacions Biomèdiques August Pi i Sunyer (IDIBAPS), UB, Barcelona, Spain.; 11 Autonomous UB, Faculty of Medicine, Unit of Anatomy and Embryology, Barcelona, Spain.; 12 University of Western Australia, Faculty of Medicine, Discipline of Medical Sciences and Genetics, School of Biomedical Sciences, Perth, Australia.; 13 Immunology Department–CDB, Hospital Clinic de Barcelona, IDIBAPS, UB, Barcelona, Spain.; 14 Bioinformatics Unit, Institut d’Investigació Biomèdica de Bellvitge – IDIBELL, L’Hospitalet de Llobregat, Spain.; 15 Institució Catalana de Recerca i Estudis Avançats (ICREA), Barcelona, Spain.; 16 Department of Molecular and Translational Medicine, University of Brescia, Piazza del Mercato, Brescia, Italy.

**Keywords:** Inflammation, Neuroscience, Neurodegeneration, Parkinson disease, iPS cells

## Abstract

Parkinson’s disease (PD) is a neurodegenerative disease associated with progressive death of midbrain dopamine (DAn) neurons in the substantia nigra (SN). Since it has been proposed that patients with PD exhibit an overall proinflammatory state, and since astrocytes are key mediators of the inflammation response in the brain, here we sought to address whether astrocyte-mediated inflammatory signaling could contribute to PD neuropathology. For this purpose, we generated astrocytes from induced pluripotent stem cells (iPSCs) representing patients with PD and healthy controls. Transcriptomic analyses identified a unique inflammatory gene expression signature in PD astrocytes compared with controls. In particular, the proinflammatory cytokine IL-6 was found to be highly expressed and released by PD astrocytes and was found to induce toxicity in DAn. Mechanistically, neuronal cell death was mediated by IL-6 receptor (IL-6R) expressed in human PD neurons, leading to downstream activation of STAT3. Blockage of IL-6R by the addition of the FDA-approved anti–IL-6R antibody, Tocilizumab, prevented PD neuronal death. SN neurons overexpressing IL-6R and reactive astrocytes expressing IL-6 were detected in postmortem brain tissue of patients at early stages of PD. Our findings highlight the potential role of astrocyte-mediated inflammatory signaling in neuronal loss in PD and pave the way for the design of future therapeutics.

## Abstract 2

Antibody targeting of astrocyte IL-6 has potential to prevent midbrain neuronal death in Parkinson's disease.

## Graphical abstract

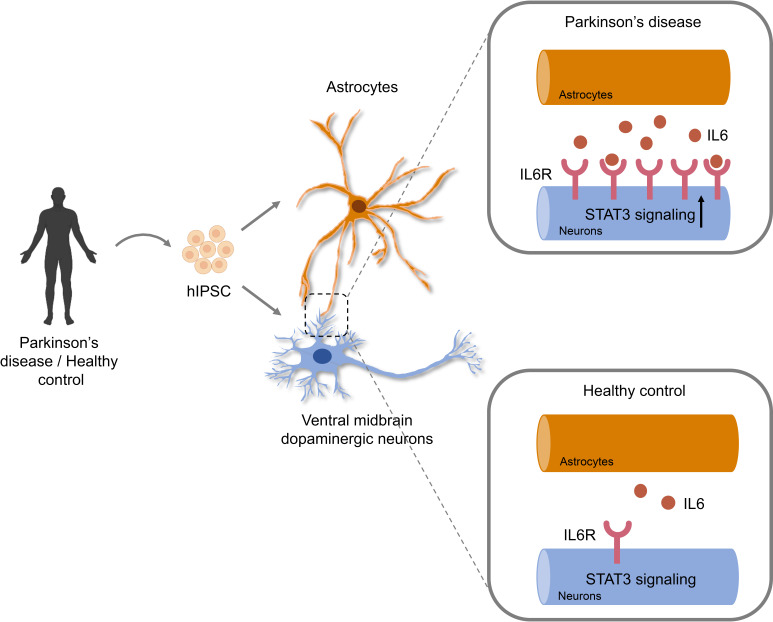

## Introduction

Parkinson’s disease (PD) is a common and incurable neurodegenerative disease associated with a progressive loss of dopamine-producing neurons (DAn) and accumulation of misfolded a-synuclein (a-syn) deposits within surviving neurons (1–3). Cumulative evidence suggests that neuroinflammation plays an important role in the pathological features and symptoms of PD (4).

Although most cases of PD, the so-called idiopathic PD (ID-PD), are of unknown cause, approximately 5% of them have been shown to have a genetic origin, with mutations in the leucine rich repeat kinase 2 (*LRRK2*) gene being the most frequent known cause of late-onset autosomal-dominant PD (L2-PD) (5). Interestingly, L2-PD closely resembles ID-PD in its neuropathological changes and responsiveness to therapy (6), suggesting that deciphering the mechanisms underlying neurodegeneration in patients with L2-PD could reveal novel therapeutic targets to treat familial PD and ID-PD.

LRRK2 is a large ubiquitously expressed protein, containing both GTPase and kinase domains involved in autophagy-lysosomal pathway (7, 8), modulation of cytoskeletal dynamics (9), vesicle trafficking, and mitochondrial function (10, 11), via binding with different interactors and increased phosphorylation of a subset of RAB GTPases (12, 13). Moreover, in the context of PD, LRRK2 expression has been identified at high levels in adaptive (14) and innate immune cells, including microglia and astrocytes (15, 16), and appears to be strongly regulated by immune stimulation (14, 15, 17–21). These findings position LRRK2 as an essential player in controlling innate immune inflammatory pathways (22), although its precise molecular and cellular function remains elusive.

Multiple lines of evidence suggest a correlation between mutant LRRK2 and several pathogenic mechanisms linked to PD initiation and progression, including an imbalanced inflammatory signaling (17). Indeed, abnormal immune activation as well as nigrostriatal dysfunction (23) have been reported in asymptomatic patients carrying the G2019S-LRRK2 mutation (24, 25) and in rodents (26–28), suggesting a close connection between LRRK2 mutations, inflammation, and disease initiation. Consistent with this, meta-analyses of human studies found elevated levels of circulating proinflammatory cytokines such as IL-6, TNF-α, IL-1b, and IL-10 in the blood of PD patients and animal models of the disease (29–31). Moreover, infiltration of peripheral immune cells, particularly T cells and monocytes, as well as the presence of activated astrocytes and microglia have been found in PD postmortem brain (32–34) or even in the early stage of the disease (35, 36).

Astrocyte dysfunction has been proposed as an active player in PD. They are the largest and most prevalent glial cell type in the CNS and are important not only for neuronal plasticity but also as regulators of the innate and adaptive immune response. Following different pathological insults, astrocytes undergo a pronounced transformation called astrocyte reactivity, characterized by both structural and biochemical changes such as high levels of glial fibrillary acidic protein (GFAP) and alterations in their immunocompetent capacity (34, 37–39). Recent observations found A1 “neurotoxic” reactive astrocytes in PD postmortem brains (34, 40). A role of astrocyte reactivity in neuronal degeneration and blood brain barrier maintenance has also been found by using human stem cell–based models (41, 42).

Previous studies, including ours, have proposed crucial non–cell-autonomous effects on neurons caused by altered LRRK2 function in glial cells. L2-PD astrocytes present dysfunctional protein degradation pathways, leading to a-syn accumulation and propagation that affects healthy DAn in an induced pluripotent stem cell–based (iPSC-based) model (43). In this line, it has been recently confirmed that L2-PD astrocytes fail to provide full neurotrophic support to DAn (44) and display atrophic morphology and altered mitochondrial function (45), metabolic alterations (46), decreased capacity to internalize a-syn (47), and downregulated genes involved in extracellular matrix (48). However, it remains unknown whether astrocyte reactivity and astrocyte-induced inflammatory mediators are altered in L2-PD astrocytes and whether they can have deleterious effects on neuronal survival.

In the current study, we sought to resolve these questions by generating and characterizing iPSC-derived astrocyte obtained from patients with ID-PD, L2-PD, and their gene-edited isogenic counterparts or from healthy individuals together with postmortem tissue brain analysis of patients with early-stage PD. Our data highlight the potential role of astrocyte-mediated inflammatory signaling in PD neuropathology.

## Results

### hiPSC-derived L2-PD astrocytes are morphologically reactive and display an altered inflammatory profile.

**Figure 1:**
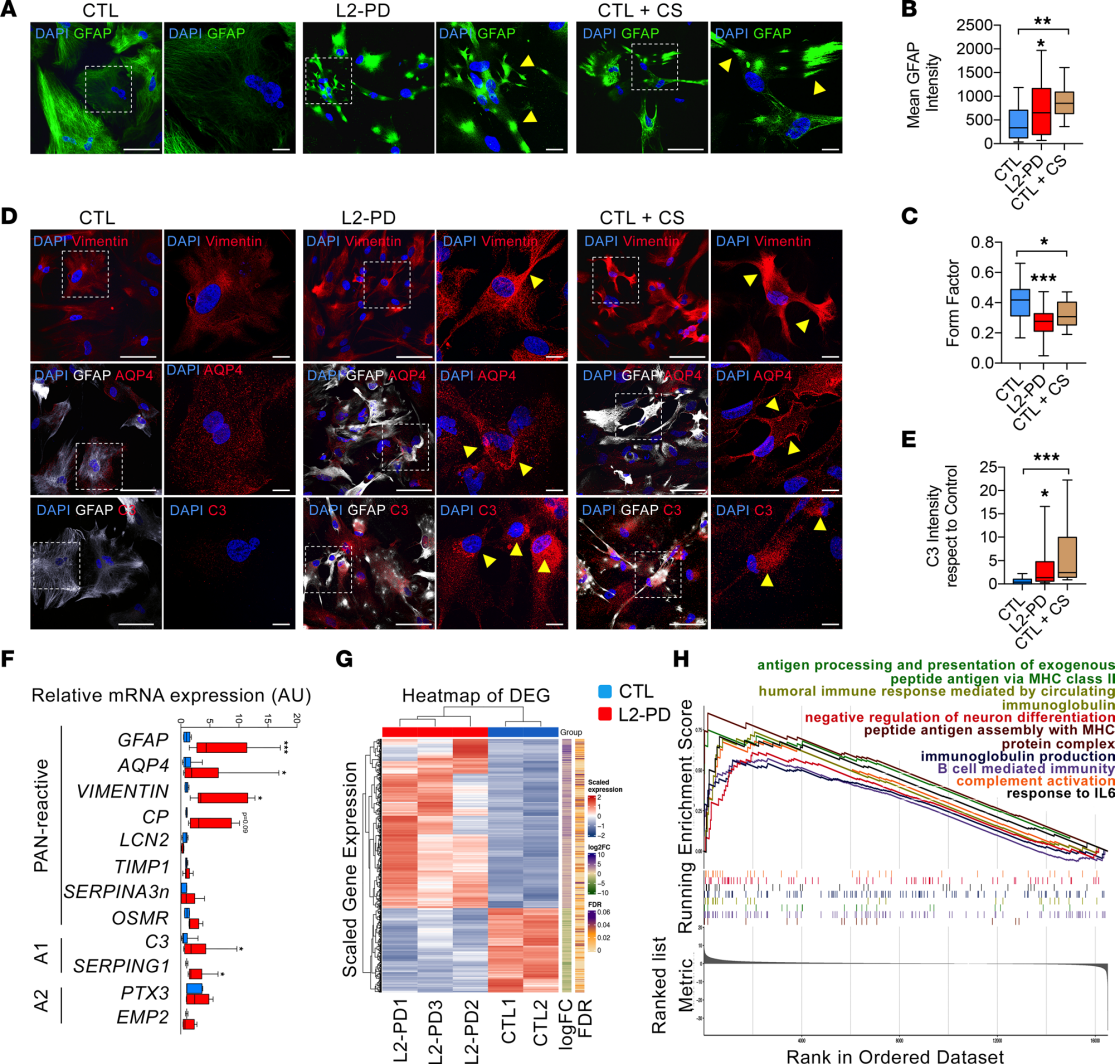
iPSC-derived L2-PD astrocytes are reactive. (**A**) Representative immunocytochemistry (ICC) images of CTL (SP09) and L2-PD astrocytes (SP13) expressing DAPI (blue) and GFAP (green) after 14 days in culture. Scale bar: 100 μm. CTL astrocytes (SP09) treated for 48 hours with C1q, TNF-α, and IL-1α were used as positive control (CTL+CS). Images on the right show a magnification of the area boxed in the left images. Scale bar: 10 μm. Yellow arrowheads indicate high GFAP staining in hypertrophic astrocytes. (**B**) Mean intensity of GFAP staining. (**C**) Form factor of GFAP^+^ cells calculated as: FF = 4 Pi number (π) (area/perimeter^2^). (**D**) Representative ICC images of CTL, L2-PD, and activated CTL astrocytes expressing Vimentin (CTL: SP09; L2-PD: SP13), AQP4 (CTL: SP17; L2-PD: SP06), and C3 (CTL: SP09; L2-PD: SP13). Scale bar: 100 μm. Images on the right show a magnification of the area boxed in the left images. Scale bar: 10 μm. Yellow arrowheads indicate high expression of the specific marker shown in the images. (**E**) Mean intensity of C3 staining with respect to CTL. (**F**) Relative mRNA expression of panreactive, A1-specific and A2-specific transcripts in L2-PD astrocytes with respect to CTL. Box-and-whisker plots show median, 25th and 75th percentiles, minimum, and maximum values (*n* = 3 experiments; form factor, mean GFAP, and C3 intensity were performed from 30 astrocytes per experiment per condition). One-way ANOVA was used, with Bonferroni as post hoc. *P < 0.05; **P < 0.01; ***P < 0.001. (**G**) Heatmap of differentially expressed genes (DEG) of CTL and L2-PD astrocytes. (**H**) Enrichment plot showing the enrichment score for the selected GO BP gene sets (e.g., antigen processing and presentation of exogenous peptide antigen via MHC class II, B cell mediated immunity, complement activation, humoral immune response mediated by circulating immunoglobulin, immunoglobulin production, negative regulation of neuron differentiation, peptide antigen assembly with MHC protein complex, response to IL-6).

We have previously described the generation of astrocytes from human iPSCs following a protocol that efficiently allows propagating, expanding, freezing and thawing proliferating glial intermediates (43, 49). Using this method, astrocytes from 3 patients with L2-PD carrying the G2019S mutation (L2-PD), 3 healthy donors (CTL), and 1 isogenic iPSC control solely differing in the correction of the mutation (L2-PD^corr^) (Supplemental Table 1; supplemental material available online with this article; https://doi.org/10.1172/jci.insight.163359DS1) were generated and cultured for 2 weeks. Interestingly, we observed that, over the 14-day time point, while CTL astrocytes maintained a large and flat morphology with low GFAP signal, L2-PD astrocytes adopted a hypertrophic morphology with thin and retracted processes as shown by GFAP staining (Figure 1, A–C, and Supplemental Figure 1A; GFAP intensity *P* < 0.05; form factor *P* < 0.001), similarly to CTL astrocytes when stimulated with a cocktail of cytokines (C1q, TNF-α, and IL-1a) (34) (Figure 1, A–C; GFAP intensity, *P* < 0.01; form factor, *P* < 0.05). Immunocytochemistry (ICC) and quantitative PCR (qPCR) of canonical markers of reactive astrocytes (“pan reactive”), such as Vimentin and AQP4 (Figure 1, D and F; *P* < 0.05), as well as A1-specific proteins such as C3 (Figure 1, D–F; *P* < 0.05), confirmed an overexpression of those markers, indicating that L2-PD astrocytes are reactive.

To evaluate if L2-PD astrocytes have altered inflammation-driven signaling that may play a role in the early stage of L2-PD, we pursued an unbiased transcriptional analysis using next-generation deep RNA-Seq to identify altered signaling pathways in PD-astrocytes. Normalized read counts of 16,775 genes were visually explored (Supplemental Table 2). The scatterplot of the first 2 principal components (PC) when considering all available genes shows that, while PC1 (capturing almost 42% of the whole variability) is not able to clearly differentiate L2-PD and CTL sample groups, PC2 (capturing almost 31% of the whole variability) separates both groups (Supplemental Figure 1B).

We found 540 differentially expressed genes (DEGs) (adjusted *P* value [*P*_adj_] < 0.05 and |log_2_ (fold change)| > 1) when comparing L2-PD against CTL samples (Figure 1G). Among them, 411 (76%) were upregulated, and the rest, 129 (24%), were downregulated (Supplemental Table 2).

The top 10 DEGs as well as validated genes by qPCR are shown in the volcano plot (Supplemental Figure 1C). To identify biological processes consistently affected across multiple L2-PD iPSC-derived astrocytes, we next conducted a gene set enrichment analysis (GSEA) with the preranked list of genes against the 7,763 gene sets derived from the Gene Ontology (GO) Biological Processes (BP) available in the collection C5 from the Human MsigDB Collections. GSEA results revealed a total of 192 significant gene sets (*P*_adj_ < 0.1). Among them, 179 of 192 (93%) were upregulated and 13 of 192 (7%) were downregulated in L2-PD astrocytes compared with CTL (Supplemental Table 2). In order to reduce the redundancy and provide a better interpretation, GSEA results were simplified by building an enrichment map based on the significant gene sets and applying an *mclust* clustering approach based on GO similarity matrix computed with the Jiang measure (Supplemental Table 2). The resulting annotated enrichment map suggests clear hubs of biological processes such as the ones called “phase cycle transition cell,” “regulation anaphase sister chromatid,” or “g2 checkpoint signaling integrity” (Supplemental Figure 1D). Among these clusters, we found the overrepresentation of inflammation-related pathways in L2-PD versus CTL iPSC-derived astrocytes included in 2 clusters named “antigen processing presentation exogenous” and “vascular endothelial growth factor.” These clusters comprise the peptide antigen assembly with MHC protein complex, antigen processing, and presentation of exogenous peptide antigen via MHC class II, B cell–mediated immunity, immunoglobulin production, humoral immune response mediated by circulating immunoglobulin, complement activation, response to IL-6, and negative regulation of neuron differentiation (Figure 1H and Supplemental Table 2), consistently with the proposed role of LRRK2 as modulator of brain immune response.

Together, these results show that L2-PD astrocytes have a different transcriptional profile compared with CTL astrocytes and display altered cytokine and immune response–related genes that might contribute to PD pathogenesis.

**Figure 2:**
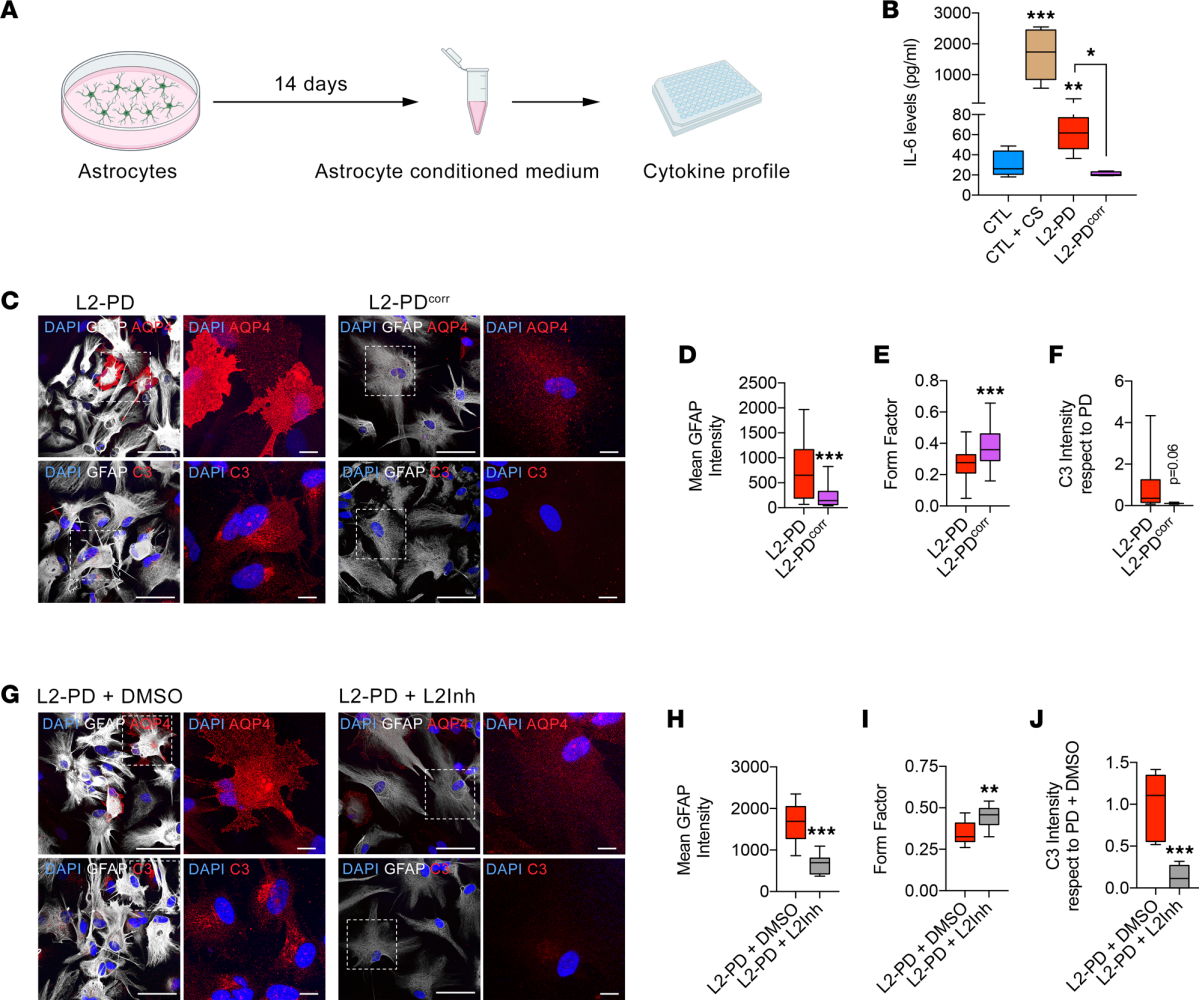
L2-PD astrocyte reactivity is mediated by LRRK2 G2019S–increased kinase activity. (**A**) Schematic experimental set up to perform a cytokine array from astrocyte conditioned medium (ACM). (**B**) Cytokine IL-6 levels released by CTL, L2-PD, and isogenic (L2-PD^corr^) astrocytes after 2 weeks in culture. CTL astrocytes were treated for 48 hours with C1q, TNF-α, and IL-1α as positive control. (**C**) Representative ICC images of L2-PD (SP13) and L2-PD^corr^ (SP13wt/wt) astrocytes staining positive for: top panels, DAPI (blue), GFAP (white), and AQP4 (red); bottom panels, DAPI (blue), GFAP (white), and C3 (red). Scale bar: 100 μm. Images on the right show a magnification of the area boxed in the left images. Scale bar: 10 μm. (**D**) Mean intensity of GFAP staining. (**E**) Form factor of GFAP^+^ cells calculated as: FF = 4 pi (area/perimeter^2^). (**F**) Mean intensity of C3 staining with respect to L2-PD astrocytes. (**G**) Representative ICC images of L2-PD (SP13) treated with either DMSO or LRRK2-kinase inhibitor (1 μM) astrocytes staining positive for: top panels, DAPI (blue), GFAP (white), and AQP4 (red); bottom panels, DAPI (blue), GFAP (white), and C3 (red). Scale bar: 100 μm. Images on the right show a magnification of the area boxed in the left images. Scale bar: 10 μm. (**H**) Mean intensity of GFAP staining. (**I**) Form factor of GFAP^+^ cells calculated as: FF = 4 pi (area/perimeter^2^). (**J**) Mean intensity of C3 staining with respect to L2-PD astrocytes treated with DMSO. Box-and-whisker plots show median, 25th and 75th percentiles, minimum, and maximum values (*n* = 3 experiments; form factor, mean GFAP, and C3 intensity were performed from 30 astrocytes per experiment per condition). One-way ANOVA was used with Bonferroni as post hoc. Student *t* test or Mann-Whitney *U* test for nonparametric conditions were used when only 2 groups were compared. *P < 0.05; **P < 0.01; ***P < 0.001.

Given the relevance of altered cytokines and immune response–related genes in L2-PD, we next examined their secretome cytokine profile. We found that, while most secreted cytokines were at comparable levels between CTL and L2-PD, IL-6 was significantly higher in the culture medium of unstimulated L2-PD astrocytes as compared with CTL and was similar to CTL cytokine-stimulated astrocytes (Figure 2, A and B [*P* < 0.01 and *P* < 0.001], and Supplemental Figure 2A), suggesting a specific upregulation of IL-6 in L2-PD astrocytes. Importantly, IL-6 was significantly reduced to CTL levels in L2-PD^corr^ astrocytes (Figure 2, A and B; *P* < 0.05), indicating that the neuroinflammatory effects depend on the LRRK2 G2019S mutation. Consistent with our cytokine profiling data, we found that L2-PD^corr^ astrocytes did not show altered morphology, nor increased GFAP, AQP4, or C3 staining, through ICC analysis (Figure 2, C–F; *P* < 0.001) or mRNA expression levels (Supplemental Figure 2B; *P* < 0.05). These findings corroborate the clinical evidence that LRRK2 is linked to inflammation (22, 23) and identify reactive astrocytes as an important player in IL-6–mediated signaling.

Next, to test whether LRRK2-kinase activity is involved in astrocyte reactivity, we treated L2-PD astrocytes with a LRRK2 kinase inhibitor for 1 week. We found that inhibition of LRRK2 kinase activity in L2-PD astrocytes prevented astrocyte reactivity, as shown by reduced GFAP, AQP4, and C3 intensity (Figure 2, G, H, and J; *P* < 0.001), and increased form factor (Figure 2I; *P* < 0.01). Importantly, inhibition of LRRK2 kinase activity significantly downregulated *IL6* expression (Supplemental Figure 2C; *P* < 0.01) along with TLRs (*TLR2* and *TLR4*), inflammasome (*NLRP1*), and inflammation-pattern recognition receptors (*CD14* and *RIG1*) as compared with nontreated conditions (Supplemental Figure 2D). In contrast, overexpression of LRRK2 G2019S (pDEST51-*LRRK2* G2019S) in CTL astrocytes significantly increased *IL6* mRNA levels (Supplemental Figure 2C; *P* < 0.01). These results suggested that the induction of IL-6 expression and neuroinflammatory alterations in human L2-PD astrocytes is mediated through the LRRK2 G2019S kinase activity.

### L2-PD astrocytes induce DA neurodegeneration by IL-6/IL-6R signaling.

**Figure 3:**
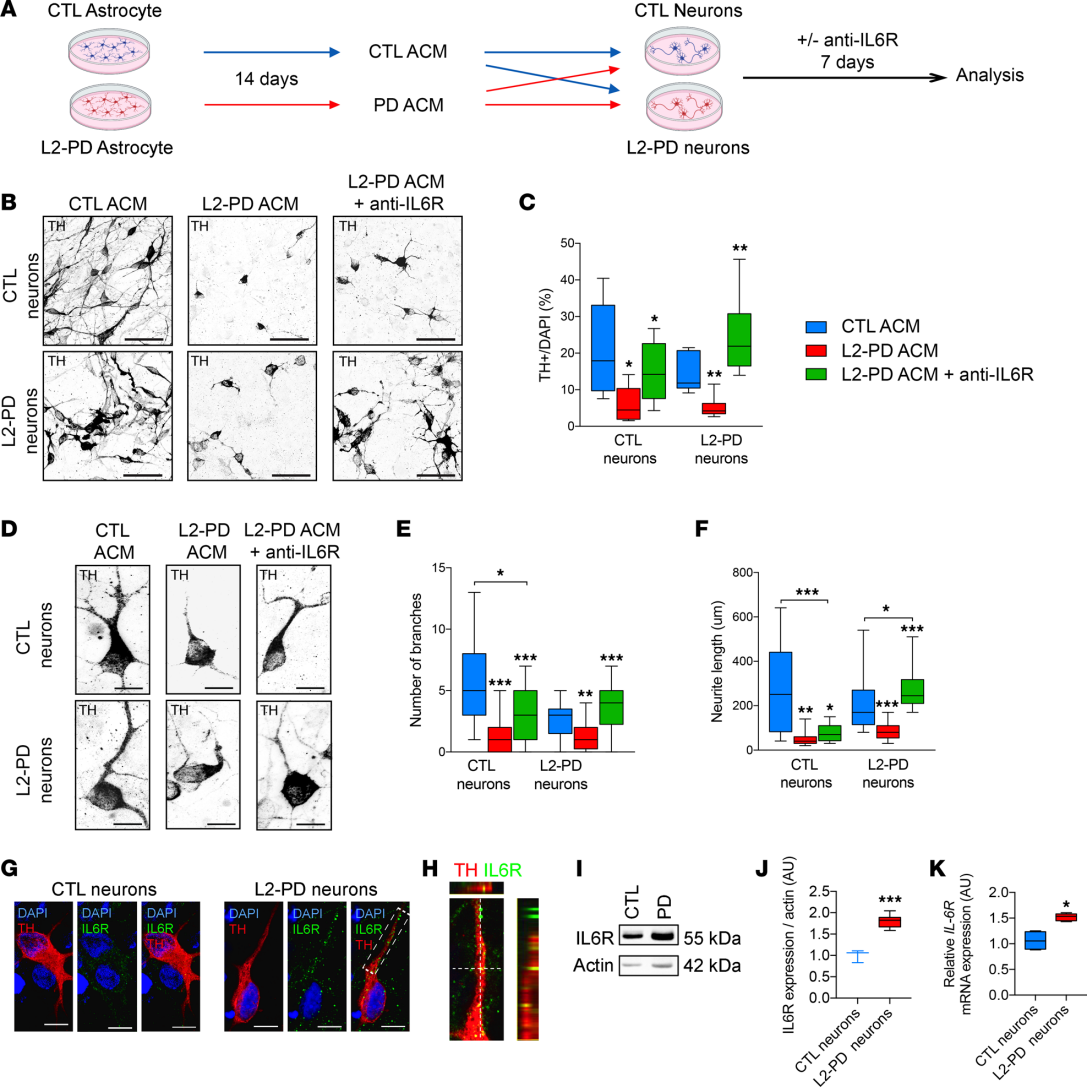
L2-PD astrocytes induce iPSC-derived DA neuronal degeneration through IL-6/IL-6R signaling. (**A**) Schematic representation of the experimental procedure to analyze the effect of astrocyte conditioned medium (ACM) and IL-6 involvement on neuronal survival and degeneration. ACM was collected after 14 days in culture and added to iPSC-derived DAn from both CTL and patients with L2-PD for 1 week. Anti–IL-6R antibody Tocilizumab (10 μg/mL) was added to ACM. (**B**) Representative ICC images of iPSC-derived CTL (SP11) and L2-PD neurons (SP12) expressing tyrosine hydroxylase (TH, black) treated for 1 week with CTL-ACM, L2-PD ACM and L2-PD ACM + anti–IL-6R antibody. Scale bar: 100 μm. CTL neurons are derived from SP11 iPSC line. L2-PD neurons are derived from SP12, SP06, and SP13 iPSC lines. (**C**) Percentage of TH^+^ cells respect to DAPI. (**D**) Representative ICC images of iPSC-derived CTL and L2-PD neurons staining for TH (black). Scale bar: 20 μm. (**E** and **F**) Number of branches and neurite length of CTL and L2-PD TH^+^ neurons treated for 1 week with ACM. (**G**) Representative ICC images of DAn (TH, red) expressing IL-6R (green) from CTL (SP11) and L2-PD (SP13) iPSC-derived DAn after 35 days of differentiation. Scale bar: 20 μm. (**H**) Orthogonal views show colocalization between IL-6R and TH in L2-PD (SP13) DAn. (**I**) Representative Western blot for IL-6R of iPSC-derived CTL neurons (SP11) and L2-PD neurons (SP12). (**J**) Quantification of protein IL-6R respect to β-actin. (**K**) Relative *IL-6R* mRNA expression from iPSC-derived CTL neurons (SP11 and SP17) and L2-PD neurons (SP12 and SP13). Box-and-whisker plots show median, 25th and 75th percentiles, minimum, and maximum values (*n* = 3 experiments; 30 neurons per experiment per condition for each line). One-way ANOVA was used with Bonferroni as post hoc (**C**, **E**, **F**). Student *t* test or Mann-Whitney *U* test (**J** and **K**) for nonparametric conditions were used when only 2 groups were compared. *P < 0.05; **P < 0.01; ***P < 0.001.

Based on the data described above, IL-6 was the only cytokine showing higher levels in L2-PD astrocyte–derived medium. We next asked whether modulation of IL-6 signaling in L2-PD or CTL DAn could be involved in neurodegeneration. To do that, we collected astrocyte conditioned medium (ACM) from astrocytes cultured for 14 days and added Tocilizumab (a humanized monoclonal antibody against IL-6 receptor (IL-6R), FDA-approved, clinically used in rheumatoid arthritis) to L2-PD–ACM and treated hiPSC-derived CTL or L2-PD neurons for 1 week (Figure 3A). In concordance with a putative IL-6 signaling, L2-PD–ACM significantly increased phosphorylated STAT3 (p-STAT3) nuclear levels in neurons (Supplemental Figure 3, A–C; *P* < 0.05) and *SOCS3* (a STAT-induced STAT inhibitor) mRNA levels (Supplemental Figure 3D; *P* < 0.001), whereas treatment with Tocilizumab reduced nuclear p-STAT3 and *SOCS3* mRNA levels to CTL-ACM levels (Supplemental Figure 3, A–D; *P* < 0.05 and *P* < 0.001). Moreover, the chronic addition of IL-6 in CTL-ACM to CTL DAn induced neurodegeneration in a concentration-dependent manner, as shown by reduced survival of Tyrosine Hydroxylase^+^ (TH^+^) neurons (Supplemental Figure 3, F–H; *P* < 0.05) and neurite arborization (Supplemental Figure 3, I and J; *P* < 0.001), whereas the blockade of IL-6 signaling through the addition of Tocilizumab prevented these alterations in DAn (Supplemental Figure 3, G–J; *P* < 0.001). These results highlight the role of IL-6/IL-6R signaling in astrocyte-neuron communication in PD.

Addition of L2-PD–ACM to DAn significantly affected DAn survival, as revealed by a reduced number of TH^+^ cells (Figure 3, B and C; CTL neurons, *P* < 0.05; L2-PD neurons, *P* < 0.01) and increased expression of *Caspase 3* mRNA levels (Supplemental Figure 3E; *P* < 0.05). Moreover, surviving TH^+^ cells displayed morphological alterations, including fewer and shortened neurites (Figure 3, D–F; *P* < 0.01 and *P* < 0.001). Remarkably, Tocilizumab treatment for 1 week promoted DAn survival and prevented neurodegeneration induced by L2-PD–ACM (Figure 3, B–F, and Supplemental Figure 3E) to both CTL and L2-PD neurons. However, the effect was significantly more pronounced in L2-PD neurons as compared with CTL, as shown by higher percentage of TH^+^ neurons (Figure 3, B and C; CTL neurons, *P* < 0.05; L2-PD neurons, *P* < 0.01) and increased neurite complexity (Figure 3, D–F; CTL neurons, *P* < 0.05; L2-PD neurons, *P* < 0.001). Together these data show a link between the mechanism responsible for neurodegeneration of L2-PD DAn and IL-6 signaling via IL-6R, and they shed light on the putative use of specific IL-6 blockers to significantly prevent DAn neurodegeneration in PD.

### L2-PD neurons are susceptible to IL-6 signaling.

After observing that L2-PD neurons are more susceptible than CTL neurons to IL-6–induced cell death, we evaluated whether there were any differences in the expression of IL-6R in L2-PD DAn versus CTL DAn. Our results show that this receptor was present in both CTL and L2-PD iPSC–derived DAn, as judged by immunofluorescence (IF) analyses (Figure 3, G and H), though quantification of IL-6R mRNA and protein levels identified higher expression in L2-PD DAn compared with CTL DAn (Figure 3, I–K [*P* < 0.05 and *P* < 0.001], and Supplemental Figure 3K). Moreover, L2-PD neurons significantly induced higher IL-6R downstream signaling effectors, as shown by the nuclear translocation of p-STAT3 along with increased STAT3 protein expression after the addition of L2-PD ACM, compared with CTL DAn (Supplemental Figure 3, L–N; *P* < 0.05 and *P* < 0.01). These results suggest that the increased expression of IL-6R in PD DAn may explain specific neuronal susceptibility to IL-6 and, therefore, to IL-6 secreted by PD-derived astrocytes, which are major producers of IL-6. Notably, the prevention of PD astrocyte–mediated neuronal degeneration, through specific blockage of IL-6R, points toward potential immunotherapeutic approaches for PD.

### IL-6/IL-6R mediates neuronal degeneration in patients with ID-PD both in vitro and in situ.

**Figure 4:**
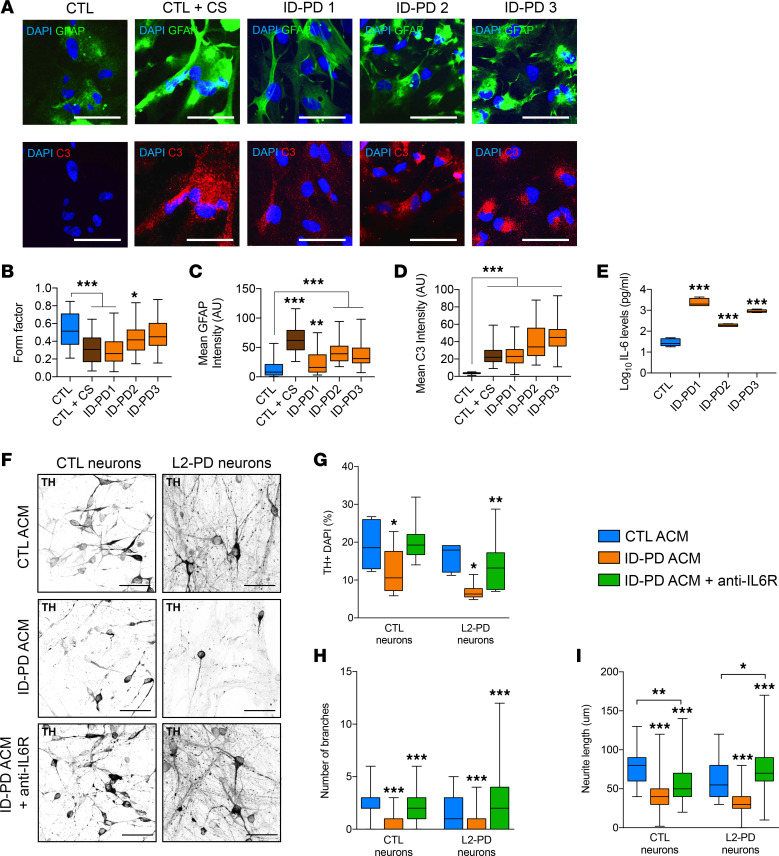
IL-6 signaling mediates neuronal degeneration in patients with ID-PD. (**A**) Representative ICC images of CTL (SP09) or ID-PD (ID-PD1: SP04; ID-PD2: SP08; ID-PD3: SP16) astrocytes staining positive for: top panels, DAPI (blue) and GFAP (green); bottom panels, DAPI (blue) and C3 (red). CTL astrocytes treated for 48 hours with cytokines were used as positive control. Scale bar: 100 μm. (**B**) Form factor of GFAP^+^ cells calculated as: FF = 4 pi (area/perimeter^2^). (**C**) Mean intensity of GFAP staining. (**D**) Mean intensity of C3 staining with respect to CTL. (**E**) IL-6 protein levels released by CTL and ID-PD ACM after 2 weeks in culture. Box-and-whisker plots show median, 25th and 75th percentiles, minimum, and maximum values (*n* = 3 experiments; form factor, mean GFAP, and C3 intensity were performed from 30 astrocytes per experiment per condition). (**F**) Representative ICC images of iPSC-derived CTL (SP11) and L2-PD (SP12) neurons expressing tyrosine hydroxylase (TH, black) treated for 1 week with CTL astrocyte conditioned medium (ACM), idiopathic (ID-PD) ACM, and ID-PD ACM + anti–IL-6R Tocilizumab (10 μg/mL). Scale bar: 100 μm. (**G**) Percentage of TH^+^ cells respect to DAPI. (**H** and **I**) Number of branches and neurite length of TH^+^ neurons cultured with ACM for 1 week. Box-and-whisker plots show median, 25th and 75th percentiles, minimum, and maximum values (*n* = 3 experiments; 30 neurons per experiment per condition for each line). One-way ANOVA was used with Bonferroni as post hoc. *P < 0.05; **P < 0.01; ***P < 0.001.

Our next goal was to investigate whether these findings also apply to patients with idiopathic forms of the disease. For this purpose, we generated astrocytes from 3 different patients with ID-PD with no familial history of PD (Supplemental Table 1). We obtained ~95% of astrocyte purity, as shown by the expression of GFAP, S100b, and CD44, and the absence of TUJ1 as well as MAP2 and NG2 (neuronal and oligodendrocyte markers, respectively) (Supplemental Figure 4, A and B). Similarly to familial L2-PD, whereas CTL astrocytes maintained a large and flat morphology with low GFAP signal, ID-PD–derived astrocytes over the 14-day time point displayed a hypertrophic and ramified morphology (Figure 4, A and B; *P* < 0.001), overexpressed GFAP and C3 at protein levels (Figure 4, C and D; *P* < 0.01 and *P* < 0.001), and upregulated inflammatory-related genes (Supplemental Figure 4C; *P* < 0.05 and *P* < 0.001), comparable with CTL astrocytes stimulated with cytokines. These results suggest that iPSC-derived astrocytes from patients with ID-PD assume a reactive phenotype, characteristic of neuroinflammatory reactive astrocytes. Moreover, we found that ID-PD astrocytes consistently release higher levels of IL-6 compared with unstimulated CTL astrocytes (Figure 4E; *P* < 0.01). To examine whether IL-6 could also be involved in astrocyte-dependent neurotoxicity in ID-PD, we added Tocilizumab to the idiopathic ACM (ID-PD ACM) and treated CTL and L2-PD neurons for 1 week. Addition of ID-PD ACM significantly reduced the number of TH^+^ cells (Figure 4, F and G; *P* < 0.05) as well as their neurite complexity (Figure 4, H and I; *P* < 0.001). Importantly, the blockade of IL-6 signaling prevented the appearance of the typical signs of neurodegeneration in ACM-treated neurons (Figure 4, F–I; *P* < 0.01 and *P* < 0.001), thus reinforcing the idea that IL-6 is one of the major mediators of astrocyte-dependent neurotoxic factors that play a role in the pathogenesis of PD.

**Figure 5:**
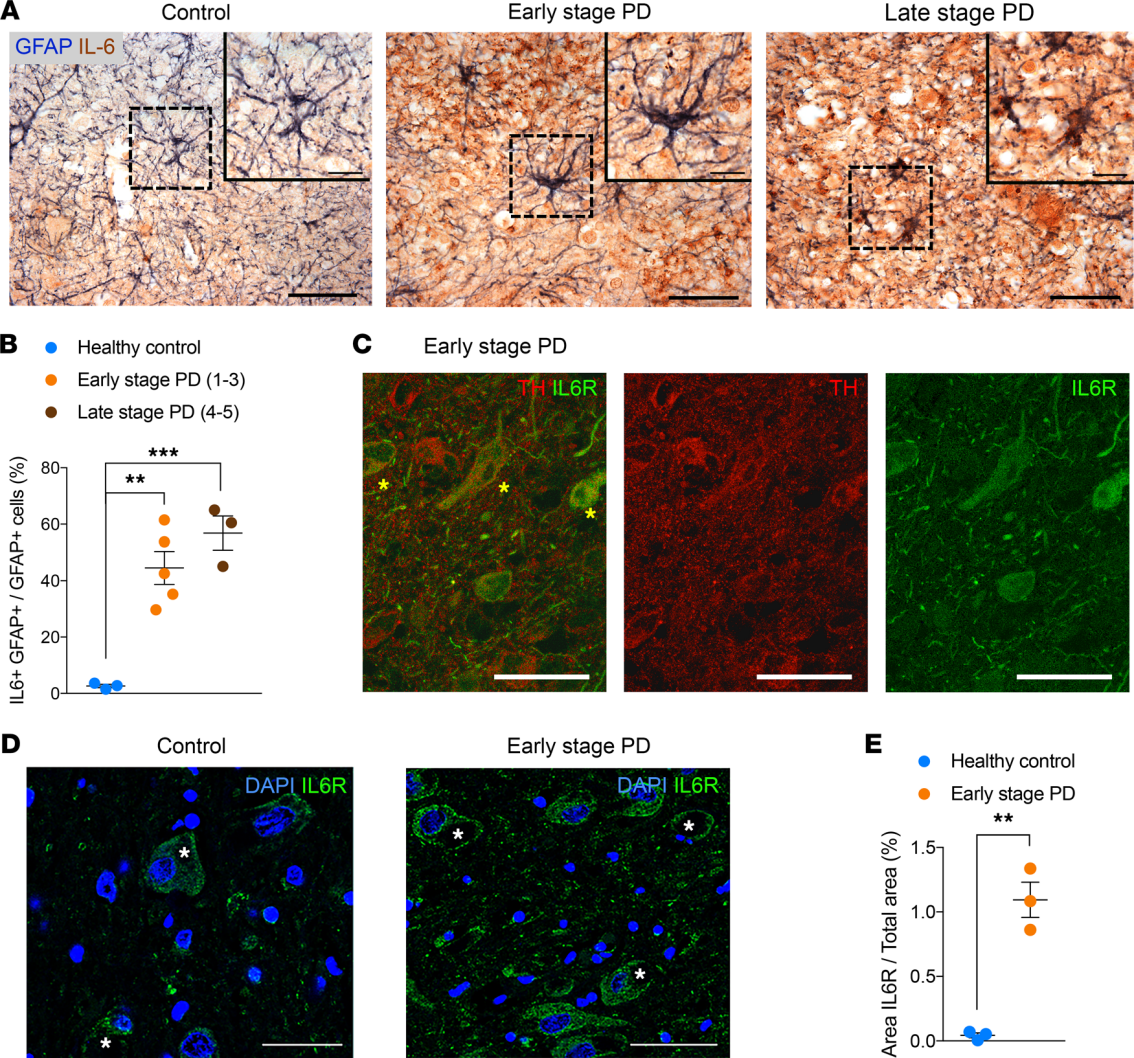
IL-6/IL-6R signaling is increased in the SNc in postmortem brains at early stages of PD. (**A**) Representative immunohistochemical sections of the mesencephalon including the substantia nigra pars compacta from control and PD cases at early (Braak stages 1–3) and late stages (Braak stages 4 and 5) of the disease. IL-6 (visualized with DAB) is present in astrocytes (GFAP^+^ cells; nickel). Scale bar: 25 μm. Images on the upper right show a magnification of the area boxed in the main images. Scale bar: 10 μm. (**B**) Percentage of GFAP^+^ cells expressing IL-6 over total GFAP^+^ cells. (**C**) IL-6R (green) is localized in DAn in PD cases (Braak stage 2) as visualized by TH immunofluorescence (red); autofluorescence is blocked by preincubating the sections with Sudan black. Scale bar: 100 μm. Asterisks show colocalization of TH^+^ cells expressing IL-6R. (**D**) Representative immunofluorescence images showing increased IL-6R immunoreactivity in PD substantia nigra pars compacta (Braak stage 2) as compared with controls. Scale bar: 25 μm. Asterisks show neurons with IL-6R expression. (**E**) Percentage of IL-6R^+^ staining area per image/total image area. Postmortem brain samples include: 3 healthy donors, 1 PD (Braak stage 1), 2 PD (Braak stage 2), 2 PD (Braak stage 3), 1 PD (Braak stage 4), and 2 PD (Braak stage 5). We counted 50 astrocytes per individual (GFAP^+^) from 6–8 images each. One-way ANOVA was used with Bonferroni as post hoc. Student *t* test was used when only 2 groups were compared. **P < 0.01; ***P < 0.001.

We next validated our findings by analyzing the presence of IL-6 and IL-6R in PD postmortem brain tissue at Braak stages 1–3 (early), stages 4 and 5 (late), and healthy controls (Figure 5). Our analysis of postmortem substantia nigra pars compacta (SNc) sections confirmed the presence of GFAP^+^ cells expressing IL-6, already at early stages of the disease. Quantifying the proportion of GFAP^+^ astrocytes expressing IL-6 over all GFAP^+^ astrocytes revealed a dramatic increase from 2.62% ± 0.612 % in controls to 44.54% ± 5.846 % in early PD stages and 56.86% ± 6.033 % in late PD stages (Figure 5, A and B; *P* < 0.01 PD early-stage versus CTL; *P* < 0.001 PD late-stage versus CTL). Interestingly, we also found larger neurons identified as DAn by colocalization with TH staining that expressed IL-6R at Braak stage 1–3 (Figure 5C). Indeed, even at early stages of PD, the SNc showed much higher levels of IL-6R expression than that of control SNc (1.09% ± 0.14 % of IL-6R^+^ staining area per image/total image area in PD versus 0.04% ± 0.02 % in controls; Figure 5, D and E; *P* < 0.001), supporting the concept that reactive astrocytes producing elevated IL-6 levels and neuronal susceptibility to IL-6 play roles during PD pathogenesis.

Overall, our findings including in vitro and postmortem data represent a direct indication that glial-related neuroinflammatory responses are key components of PD physiopathology and may have therapeutic implications for future intervention in early stages or during the course of PD.

## Discussion

We have previously demonstrated that non–cell-autonomous effects on neuronal cells are mediated by LRRK2-PD (L2-PD) astroglial–a-syn in our iPSC-based model of PD (43). While these data provide direct evidence of how dysfunction of astrocytes carrying the G2019S LRRK2 mutation can lead to neurodegeneration associated with PD, whether the specific mutation altered the inflammatory profile of the corresponding PD astrocytes had not been examined.

In this study, by using our previously established human model of PD (43), we derived astrocytes from 3 L2-PD patient-specific iPSC and 3 control iPSC, and we showed that L2-PD astrocytes present a unique proinflammatory cytokine profile and transcriptional inflammatory-related pathways. Notably, we found that L2-PD astrocytes are transcriptionally distinct from controls and exhibit features of reactive astrocytes, such as overexpression of GFAP and C3, even without stimulation. GO analysis revealed upregulated response to IL-6 signaling and complement activation in L2-PD astrocytes compared with CTL. These phenotypes are accompanied by a specific overproduction and release of IL-6, by a more than 2.5-fold increase in L2-PD astrocytes, and appear to be mediated by hyperactive LRRK2 G2019S kinase activity, corroborating the proposed role of LRRK2 as modulator of brain immune response.

Mechanistically, we show that IL-6 signaling, via IL-6R expressed in human neurons, leads to downstream activation of STAT3, mediating DA neurodegeneration, accordingly with previous data showing increased levels of nuclear pSTAT3 in the SNc of PD mouse models (50). Conditioned culture medium of L2-PD astrocytes, exposed for 7 days to L2-PD or control neurons, was sufficient to induce neuronal cell death, and this effect was prevented in the presence of an IL-6R blocking antibody. Thus, IL-6 secreted by L2-PD astrocytes directly and negatively affected neuronal survival, suggesting that L2-PD reactive astrocytes are key mediators of neuroinflammatory responses that lead to DAn degeneration in PD.

Importantly, we found that human L2-PD neurons are more susceptible than CTL neurons to IL-6–induced cell death and that they express higher levels of IL-6R, suggesting that this susceptibility can be related to the increased expression of IL-6R in their membrane. Blocking IL-6 signaling in a purely PD context (PD neurons/PD astrocyte) with a specific antibody FDA-approved (Tocilizumab) prevented neuronal degeneration significantly more compared with control neurons treated with PD-ACM. Although the mechanisms by which IL-6R is upregulated in PD remain unknown, it is possible to speculate that IL-6R membrane trafficking could be altered in PD, leading to its accumulation, since LRRK2 has been widely involved in vesicle trafficking and endocytosis (10, 11). Thus, the use of Tocilizumab to prevent IL-6R–related changes in DAn suggests that antibody-mediated blockade/neutralization of IL-6 represents a viable therapeutic strategy to treat PD with a significant translational effect in the design of future therapeutics.

IL-6 can be produced by astrocytes, microglia, and neurons, following CNS injury and neuroinflammatory response. Elevated levels of IL-6 have been found in the serum, cerebrospinal fluid, and SNc of patients with PD already at Braak stages 1 and 2 (51, 52), and higher IL-6 levels have been correlated with a worse PD prognosis (53, 54), suggesting that IL-6/IL-6R signaling can be an important therapeutic target. As a key target of inflammation, IL-6 has been studied in various systems revealing multiple pleiotropic effects, including its description as a founding member of neuropoietins and its proinflammatory roles in CNS-related diseases (55, 56). Indeed, while acutely in vitro, it acts as a neuroprotectant; however, chronic treatment has been associated with neuronal cell injury and death (57–59). The results of our study show that overproduction of IL-6 has a detrimental effect on DAn, most likely due to synaptic damage and reduced connectivity between neurons, as previously described for a complement system (60).

The observation that L2-PD astrocytes are reactive and release IL-6 without being in contact with PD neurons suggests that the astrocyte- inflammatory profile depends on the LRRK2 G2019S mutation. Indeed, L2-PD^corr^ astrocytes, derived from the same patient but in which the mutation has been corrected, as well as the use of a LRRK2 kinase inhibitor significantly restored inflammatory-dependent phenotypes to unstimulated CTL levels. Previous studies support a role for LRRK2 as a regulator of inflammation in microglia cells both in vitro and in mice carrying LRRK2 mutations (25, 26, 61) as well as in mutant LRRK2 lymphocytes where increased release of IL-6 has been suggested as an inducer of neurodegeneration (62). The mechanisms by which LRRK2 regulates proinflammatory IL-6 is unknown, although LRRK2 has been proposed as a regulator of NF-κB and NFAT transcription factors (63, 64). However, considering the functions of a complex kinase such as LRRK2, which is expressed in multiple cell types, we cannot exclude that the present described mechanism could be one of the multiple mechanisms underlying the pathological processes occurring in PD.

Notably, we observed increased levels of IL-6 in ID-PD reactive astrocytes in vitro as well as an increased number of DAn overexpressing IL-6R, with correlative increases in reactive astrocytes expressing IL-6 in postmortem brain tissue of early- and late-stage of PD, compared with healthy brains. These data reinforce the notion that neurotoxic levels of IL-6 could be a common mechanism in PD pathology. Although the mechanisms by which IL-6 is increased in ID-PD astrocytes remains unknown, recent studies point to the presence of hyperactivated kinase LRRK2 in patient cells (65). Moreover, in line with our results, PD was one of the first neurodegenerative disorders to our knowledge in which inflammatory/reactive A1-like astrocytes were initially identified in postmortem SNc tissue samples (34).

Apart from reactive astrocytes, postmortem samples displayed significant levels of inflammatory microglia activation (66). Microglia and astrocytes are glial cells that interact with and support neurons and are involved in the immune reaction in response to brain injury or disease (67–70). Similar to astrocytes, in response to pathological situations in their surroundings, microglia can become reactive, a state characterized by altered functions and an amoeboid morphology as well as the release of pro- and antiinflammatory cytokines (67, 69–71). Since PD is a chronic condition, it stands to reason that both microglia and astrocytes may be involved in the neuronal degeneration that occurs in PD. Given that our current model is composed only of iPSC-derived astrocytes and neurons, adding complexity to our culture system via the study of microglia and the interaction between these 2 glial cell types would be useful in the investigatation of the overall contribution of these signaling events in PD and the effect of microglia and astrocyte inflammatory signaling toward DAn cell death

Nevertheless, our study reveals the contribution of glia-related neuroinflammatory responses to the pathophysiology of PD and indicates the blockade/neutralization of IL-6R antibodies as a possible and valid therapeutic strategy to prevent, slow down, or possibly halt PD pathogenesis. Considering that IL-6 may be involved in chronic inflammation in PD, our study identifies a possible therapeutic approach in PD by an immune modulation of inflammatory cytokines.

## Methods

### iPSC-derived astrocyte generation and culture

The parental iPSC lines used in this study were previously generated and fully characterized (72). Specifically, we used 3 iPSC lines obtained from 3 patients carrying the LRRK2 G2019S mutation (L2-PD1: SP06, L2-PD2: SP12, and L2-PD3: SP13) and from 3 healthy age-matched controls (CTL1: SP09, CTL2: SP17 and CTL3: SP11-FLAG). The isogenic control (L2-PDcorr: SP13 wt/wt), was previously obtained by correcting the LRRK2 mutation in the SP13 iPSC line (43).

Astrocytes were generated from patient-derived iPSC as previously described (43, 49). Briefly, iPSCs were cultured and differentiated into spherical neural masses (SNMs) that were pushed toward an astrocytic lineage. First, SNMs were grown in suspension for 28 days with induction medium (DMEM/F12 [Invitrogen], 1% N-2 supplement, 0.1% B27 supplement [Invitrogen, 17504-044], 1% nonessential amino acids [NEAA; Corning], 1% penicillin/streptomycin [PenStrep; PS-B LabClinics], 1% glutamax [Invitrogen]) supplemented with 20 ng/mL LIF (MilliporeSigma) and 20 ng/mL EGF (R&D Systems), and again for a further 21 days with propagation medium (DMEM/F12, 1% N-2 supplement, 0.1% B27 supplement, 1% NEAA, 1% PenStrep, 1% glutamax) containing 20 ng/mL FGF-2 (PeproTech) and 20 ng/mL EGF (R&D Systems). Finally, SNMs were dissociated into a monolayer, plated on matrigel-coated plates, cultured for 14 days in propagation medium and then for another 14 days in CNTF medium (Neurobasal [Invitrogen], 1% Glutamax, 1% PenStrep, 1% NEAA, 0.2% B27 supplement, 10 ng/mL CNTF [Prospec Cyt-272]). Experiments were performed with astrocytes growing on Thermanox plastic coverslips (Thermo Fisher Scientific) coated with matrigel (Corning) in 24-well plates or in 6-well plates for RNA analysis.

ACM was obtained as follows: 2.5 × 10^5^ astrocytes were plated per well on a matrigel coated 6-well plate in 2 mL of CNTF medium. Each line was cultured for 14 days without changing the initial medium. At day 6, 1 mL of fresh CNTF medium was added to each well. After the 14-day time point, the medium was collected and frozen at –80°C.

### iPSC-derived vmDAn generation

Four different iPSCs, 3 L2-PD (SP06, SP12 and SP13) and 1 CTL (SP11), were differentiated into DAn using the 3-step system PSC Dopaminergic Neuron Differentiation Kit (Thermo Fisher Scientific) according to the manufacturer’s protocol. Briefly, iPSCs were maintained in conditioned HES medium until they reached 70% of confluence and were then passaged with EDTA (Invitrogen) to Vitronectin-coated plates (Thermo Fisher Scientific; 10 μg/mL) with 3.0 × 10^4^ viable cells/cm^2^ in conditioned HES medium (Invitrogen) with 10 μM ROCK inhibitor (Miltenyi Biotec; Y27632; RI). The day later, step 1 began by changing the medium to Complete Floor Plate Specification Medium (Neurobasal, 20× Floor Plate Specification Supplement, 1% PenStrep) for up to 10 days, with a complete medium change every 2 days to move iPSC into midbrain-specified floor plate progenitor (FP) cells. On day 10, the second step of ventral midbrain DAn (vmDAn) differentiation started with the expansion of FP cells as adherent cultures in Complete Floor Plate Cell Expansion Medium (Floor Plate Cell Expansion Base Medium, 50× Floor Plate Cell Expansion Supplement, 1% PenStrep) on laminin-coated plates (Sigma-Aldrich; L2020; 3 μg/mL) for 2 passages on days 12 and 16 (FPp1 and FPp2, respectively) with Accutase (Invitrogen) to a 1:4–6 split ratio onto laminin-coated plates with 5 μM of RI. Medium was fully changed every 2 days. On day 16, FPp2 could be either frozen with FP freezing media (90% Floor Plate Cell Expansion Medium and 10% DMSO [Sigma-Aldrich; D2438]) or expand the FP progenitors in sphere formation transferring cells to low-attachment plastic culture plates in Floor Plate Cell Expansion Medium, with a complete medium change every 2 days. Once the spheres were formed by day 21, they could be either frozen with FP freezing media or dissociated, following the step 3 for vmDAn maturation. Dissociated cells were plated into Poly-D-lysine (Thermo Fisher Scientific; A3890401) and Laminin double-coated plates (100 μg/mL-3 μg/mL) in Complete Maturation Medium (Neurobasal, 50× Dopaminergic Neuron Maturation Supplement, 1% NEAA, 1% PenStrep, 1% Glutamax), with 5 μM of RI to a density of 1.0 × 10^5^ cells/cm^2^. Medium was fully changed every 2 days until day 35, during which vmDAn were used for experiments.

### Cytokine array

The HCYTOMAG-60K-15 Human Cytokine/Chemokine Multiplex Assay (MilliporeSigma) was employed to simultaneously analyze TNF-α, IL-1b, IL-1a, IL-2, IL-4, IL-6, IL-10, Eotaxin, FLT3L, GMCSF, Rantes, MCP1, Fractalkine, and IFN-γ with Bead-Based Multiplex Assay using the Luminex technology. C3 and C1 were analyzed with HCMP2MAG-19K-02 Human Complement Magnetic Bead Panel 2 (MilliporeSigma). Supernatants from nonstimulated CTL and L2-PD astrocytes cultured for 14 days were collected and stored at –80°C for long storage. CTL astrocytes stimulated with C1q, TNF-α, and IL-1a served as positive controls. Quality controls (QC) and human cytokine standard (STD) were prepared with deionized water by serial dilutions according to manufacturer instructions. Briefly, wells were filled with 200 μL of wash buffer (WB), sealed, and mixed at room temperature (RT) for 10 minutes on a Thermomixer Comfort plate shaker at 500–800 rpm. The plate was firmly covered and turned upside down on absorbent paper, and 25 µL of samples of each STD, QC, and CNTF medium as negative control were added to the plate. In total, 25 μL of sample with 25 μL of assay buffer were added in duplicate. Finally, 25 μL of the mixed antibody-bead preparation was added to every well and incubated at 2°C–8°C overnight in a plate shaker. The day afterward, well contents were gently removed by firmly attaching plate to a handheld magnet and washed with WB for 3 times. Then, 25 μL of detection antibodies were added into each well and incubated at RT for 1 hour on a plate shaker. After that, 25 μL of Streptavidin-Phycoerythrin was added into each well and incubated at RT for 30 minutes on a plate shaker. Well contents were gently removed with a handheld magnet and washed with WB for 3 times. Antibody beads were resuspended by adding 150 μL of Sheath Fluid to all wells and incubated at RT for 5 minutes on a plate shaker. Finally, a plate was run on a multiplex system Luminex 200 (Invitrogen). Probe heights of every antibody were adjusted according to Luminex recommended protocols employing the xPONENT software (Luminex). Mean fluorescence intensity of every analyte was analyzed using a 5-parameter logistic or spline curve–fitting method for calculating analyte concentrations in samples.

### Cell culture treatments

LRRK2-kinase inhibitor (1 μM; MilliporeSigma, 438193) was added to astrocytes every 48 hours and fixed after 1 week. Anti–IL-6R antibody (Tocilizumab) was provided by Dr. Manel Juan Otero (Hospital Clinic, Barcelona). Tocilizumab (10 μg/mL) and IL-6 (200-06; Peprotech; 10 ng/mL) was added to neurons together with ACM every 48 hours. Neurons were fixed after 1 week of treatment.

### ICC

Samples were fixed using 4% PFA and then washed with PBS. Samples were blocked and permeabilized with blocking solution (TBS, 3% normal donkey serum [Merck], 0.01% Triton X-100 [Sigma-Aldrich]) for 2 hours and subsequently incubated with the primary antibody for 48 hours at 4°C. Primary antibodies used include rabbit anti-AQP4 (MilliporeSigma, B104662), rabbit anti-C3 (Dako, A0063), guinea pig anti-GFAP (Synaptic Systems, 173 004), rabbit anti-GFAP (Dako, Z0334), mouse anti–IL-6 (R&D systems, MAB206-SP), mouse anti–IL-6R (R&D systems, MAB2271), sheep anti-TH (Pel-Freez, P60101-0), rabbit anti-TH (MilliporeSigma, T8700), and mouse anti-Vimentin (Iowa, 40E-C). Samples were then washed 3 times with TBS and incubated with secondary antibodies (1:250) for 2 hours at room temperature: Alexa Fluor 488 anti–mouse IgG (Jackson ImmunoResearch, 715-545-150), Cy3 anti–rabbit IgG (Jackson ImmunoResearch, 711-165-152), DyLight 649 anti–guinea pig IgG (Jackson ImmunoResearch, 706-495-148), Alexa Fluor 647 anti-sheep (Jackson ImmunoResearch, 713-605-147), Cy2 AffiniPure donkey anti–rabbit igG (H+L) (Jackson ImmunoResearch, 711-225-152), and Cy3 AffiniPure donkey anti-mouse IgG (H+L) (Jackson ImmunoResearch, 715-165-151). Samples were washed with TBS 1× followed by incubation with nuclear staining DAPI (Invitrogen, 1:5,000) for 10 minutes, mounted with PVA-DABCO (MilliporeSigma), and stored at 4°C until imaged. Samples were imaged using an SPE or SP5 confocal microscope (Leica) and analyzed with FIJI using either the Cell Counter Plugin, Colocalization Plugin, or Simple Neurite Tracer Plugin. Neurite length and number of terminals were analyzed for TH^+^ cells from an average of 5 images and 10 neurons per image.

### Cellular transfection

In total, 2 × 10^4^ astrocytes seeded in 24-well plates (or 2.5 × 10^5^ in 6-well plates) were cotransfected after 7 days in culture with 1 μg pDEST51-LRRK2 G2019S, which was a gift from Mark Cookson (Addgene plasmid no. 29401) or 1 μg of GFP expression plasmid as transfection control and collected after 48 hours. Transfection was done using Lipofectamine Stem Reagent (Invitrogen) following manufacturer instructions.

### RNA extraction and gene expression analysis

The isolation of total mRNA was performed with the RNeasy Micro Kit and treated with Rnase free Dnase I (Qiagen). In total, 500 ng was used to synthesize cDNA with the SuperScript III Reverse Transcriptase Synthesis Kit (Invitrogen). qPCR analyses were done in triplicate using 2 ng/μL cDNA with Platinum SYBR Green qPCR Super Mix (Invitrogen) in an ABI Prism 7000 thermocycler (Applied Biosystems). All results were normalized to β-actin. Primers used are listed in Supplemental Table 3.

### Stranded mRNA library preparation and sequencing

Total RNA was assayed for quantity and quality using Qubit RNA HS Assay (Invitrogen) and RNA 6000 Nano Assay on a Bioanalyzer 2100. The RNASeq libraries were prepared from total RNA using the TruSeq Stranded mRNA LT Sample Prep Kit (Illumina). Briefly, 500 ng of total RNA was used as the input material and was enriched for the mRNA fraction using oligo-dT magnetic beads. The mRNA was fragmented in the presence of divalent metal cations and at high temperature (resulting RNA fragment size was 80–250 nt, with the major peak at 130 nt). The second strand cDNA synthesis was performed in the presence of dUTP instead of dTTP, and this allowed for strand specificity. The blunt-ended double-stranded cDNA was 3′adenylated, and Illumina indexed adapters were ligated. The ligation product was enriched with 15 PCR cycles, and the final library was validated on an Agilent 2100 Bioanalyzer with the DNA 7500 assay. The libraries were sequenced on HiSeq2000 (Illumina) in paired-end mode with a read length of 2 × 76 bp using TruSeq SBS Kit v4. We generated over 30 million paired-end reads for each sample in a fraction of a sequencing v4 flow cell lane, following the manufacturer protocol. Image analysis, base calling, and quality scoring of the run were processed using the manufacturer’s software Real Time Analysis (RTA 1.18.66.3), followed by generation of FASTQ sequence files by CASAVA. The RNA-Seq data have been deposited in Gene Expression Omnibus (GEO) of the National Center for Biotechnology Information and are accessible through GEO Series accession no. GSE207713.

### Bioinformatics of RNA-Seq

#### Read preprocessing, mapping, and feature counting.

Raw FASTQ files were examined using FastQC (v0.11.8) (73). Illumina adapter sequences, low quality, and possible contaminated reads were removed from raw reads with Trimmomatic (v0.36), using default settings but requiring a minimum average quality of 20 (74). Trimmed paired-end reads were mapped to the human reference genome (GRCh38_primary) using STAR v2.7.1a (75) with ENCODE parameters for long RNA. Raw counts of each annotated gene (GENCODE v30) were quantified using the featureCounts function from the Bioconductor package Rsubread (v1.34.7) (76). Genes on the resulting matrix with read counts were identified by the NCBI Entrez Gene. A complete gene annotation matrix was obtained from the GTF annotation file, which referred to 28,396 different genes. Information included the correspondence between NCBI Entrez Gene ID, Official Symbol, and Ensemble Stable Gene ID. Additionally, official full name, gene type, location (e.g., chromosome name, start and end positions on the chromosome), width of the gene, as well as aliases for the same gene data was kept. Those genes with less than 5 reads among all samples were removed. This led to a final raw counts matrix referring to 16,775 genes (Supplemental Table 2).

#### Exploration data analysis.

In-house R-scripts were used for performing an exploration data analysis. This analysis was conducted to explore characteristic patterns and identify potential undesired effects. In this regard, PC analysis (PCA), hierarchical clustering based on Pearson correlation coefficient and complete method, and read count distribution visualized in box plots were used to visually inspect the overall behavior of the samples. The logarithm transformation, regularized log transformation, and variance stabilizing Transformation feature in the Bioconductor package DESeq2 v1.24.0 (77) were applied to normalize and compare the counts matrix.

#### Differential expression analysis.

DEGs between L2-PD and CTL patients were identified suing the Bioconductor package DESeq2. The method fits a negative binomial generalized linear model (GLM) and estimates the log_2_ fold change between groups for each gene. After the fit, it performs a Wald test to assess the null hypothesis that there is no differential expression between L2-PD and CTL for each gene. Raw *P* values were adjusted for multiple testing using the Benjamini-Hochberg FDR (78). Those genes showing a *P*_adj_ lower than 0.05 and an absolute log_2_ fold change higher than 1 were considered differentially expressed (Supplemental Table 2). Volcano plots were built with the Bioconductor package EnhancedVolcano v1.16.0 (79) to visualize the overall distribution of all DEGs between L2-PD and CTL and to highlight the genes of interest (Supplemental Figure 1C).

#### Functional enrichment analysis.

To further explore the biological significance of DEGs, the gene sets derived from the GO BP provided in the collection C5 from the Broad Institute’s Molecular Signatures Database (MSigDB v3.1) (80) were downloaded to perform a functional enrichment analysis based on the GSEA approach (81, 82). The analysis was conducted with the function GSEA from the Bioconductor package clusterProfiler v4.6.0 (83, 84). Genes were preranked by –log_10_ (Supplemental Table 2). The function was parameterized with the fgsea mode, a minimum gene set size of 15, an epsilon constant equal to 0, a *P* value cutoff of 1 to generate the whole list of results, the Benjamini and Hochberg method to compute the associated *P*_adj_, the size of a random set of genes to control the estimation accuracy set up to 101, and the number of permutations in the simple fgsea implementation for the preliminary estimation of *P* values was fixed to be 25,000. Gene sets considered for the analysis were gene sets showing an *P*_adj_ lower than 0.1 and were considered statistically significant (Supplemental Table 2).

#### Simplification of functional enrichment results.

In view of simplifying GSEA results for improving the biological interpretation, several clustering methods based on different semantic similarity measures were compared. This data analysis was conducted using the Bioconductor package simplifyEnrichment v1.8.0 (85). The GO semantic similarity matrices were obtained with the function GO similarity. This function was fed with the GO accession nos. corresponding to the selected BP gene sets and the Bioconductor organism package org.Hs.eg.db v.3.16.0 (86), and it was executed for 3 different methods based on 2 functional similarity approaches. On one hand, we examined a graph-based method called Wang method (87) (Supplemental Table 2), and, on the other hand, we studied 2 information content-based methods, named Relevance (88) and Jiang (89) methods (Supplemental Table 2). For each of the 3 GO semantic similarity matrices, we compared all the available clustering methods in the function compare clustering methods. First, we used heatmaps for the specific similarity matrix under the 11 clustering methods plus a table with the number of clusters. Second, we used a figure with 3 panels showing (a) a heatmap of the similarity matrix with different clusterings provided as row annotations, (b) a heatmap of the pairwaise concordance of the clustering methods, and (c) bar diagrams of the difference scores, the number of clusters, and the mean similarity of the gene sets that are in the same clusters for each method. After visually inspecting the resulting plots, we decided to focus on the mclust method for simplifying the redundancy of the gene sets. This method showed similar and good levels of difference score, number of clusters, and block mean similarity for the 3 GO semantic similarity matrices. Therefore, to simplify the GO terms, we applied the function simplifyGO for each similarity matrix, applying the mclust clustering algorithm. The function was configured to generate a heatmap showing word cloud annotations summarizing the biological processes with keywords in every gene set cluster (Supplemental Table 2). The seed was set to 1,973 for all procedures where a random number generations procedure was involved.

#### Visualization of simplified functional enrichment results.

For the sake of clarity, we generated a network using the EnrichmentMap Cytoscape App v3.3.5 (90) with the list of GO_BP terms generated after simplifying the GSEA results. To create the enrichment map, we selected the GSEA as the analysis type, and we provided the lists of positive and negative enrichments derived from the GSEA results, the GMT file corresponding to C5 BP subsets of GO downloaded from the Human MSigDB Collections, the list of the preranked genes, and the expression matrix and the phenotype labels file. We used a FDR cut-off of 0.1 to select the significantly enriched gene sets as mentioned above, and we applied a medium connectivity. The resulting EnrichmentMap was visualized in Cytoscape v3.9.1 (91). To automatically annotate, group, and collapse the nodes, we used the AutoAnnotate Cytoscape App v1.4.0 (92) (Supplemental Figure 1D). We used the clusters defined by the mclust algorithm based on the Jiang semantic similarity matrix, and we set up the maximum words per label to 4. The resulting annotated network provided a comprehensive view of the GO gene sets enriched in the analysed data set and allowed us to identify a couple of biological process clusters associated with the DEGs. These clusters were named, by the AutoAnnotate App, “antigen processing presentation exogenous” and “vascular endothelial growth factor” corresponding to the 16 and 18 clusters identified by the mclust with the Jiang semantic similarity matrix. For these clusters, we retrieved the associated genes (Supplemental Table 2).

### Protein extraction

Cytoplasm and nucleus fractions were separated using Buffer A (10 mM HEPES, 1.5 mM MgCl_2_, 10 mM KCl, 0.5 mM DTT, 0.05% NP40 [pH 7.9]) followed by centrifugation at 1100*g* for 10 minutes at 4°C. Pellets were homogenized in Laemmli buffer (4% SDS, 10% β-mercaeptoethanol, 20% glycerol, 0.125M Tris-HCl [pH 6.8], and 0.005% of bromophenol blue) and sonicated for 10 seconds. Samples were then centrifuged at 15,000*g* for 20 minutes at 4°C. The resulting supernatant was normalized for protein using BCA kit (Pierce).

### Western blot

Cell extracts were boiled at 95°C for 5 minutes, followed by 10% SDS-PAGE, transferred to Nitrocellulose or PVDF membranes for 1.5 hours at 4°C and blocked with 5% nonfat milk in 0.1M Tris-buffered saline (pH 7.4) for 1 hour. Membranes were incubated overnight at 4°C with primary antibodies diluted in TBS/3% BSA/0.1% TWEEN (Sigma-Aldrich) or TBS 5% milk/0.1% TWEEN. After incubation with peroxidase-tagged secondary antibodies (1:2,500), membranes were revealed with ECL-plus chemiluminescence Western blot kit (Amershan-Pharmacia Biotech). The following antibodies were used: mouse anti–β-actin (Affinity, T0022), mouse anti–IL-6R (R&D Systems, MAB2271), rabbit anti–p-STAT3 (Tyr705) (Cell Signaling Technology, 9145, D3A7), rabbit anti-STAT3 (Cell Signaling Technology, 12640, D3Z2G), rabbit anti–α-tubulin (MilliporeSigma, T3526), and goat anti–Lamin B (Santa Cruz Biotechnology, sc-6217). Films were scanned at 2,400 × 2,400 dpi (i800 MICROTEK high-quality film scanner), and the densitometric analysis was performed using FIJI. Other membranes were imaged using the ChemiTouch machine under the optimal exposure setting.

### IHC and IF of human brain samples

Fixed sections of the mesencephalon in 4% PFA, including the substantia nigra, were obtained from the Institute of Neuropathology Brain Bank. Tissue samples were from 5 cases with premotor PD at Braak stages 1, 2, and 3; from 3 cases with clinical PD at Braak stage 4 and 5; and from 3 age-matched controls (equal representation of men and women; mean age 65 years in every group). Cases with concomitant neurodegenerative diseases, including Alzheimer’s disease–related changes were excluded. The postmortem delay was up to 13 hours. PFA-fixed sections, 4 μm–thick, were processed for IF, confocal microscopy, and IHC. Antibodies used were: rabbit polyclonal antibodies against IL-6 (Abcam, ab6672) diluted 1:100; rabbit polyclonal anti–IL-6R (R&D Systems, MAB2271) diluted at 1:50; anti-GFAP antibody (Diagnostic BioSystems, Mob064) diluted at 1:1,000, and rabbit anti-TH (MilliporeSigma, T8700) diluted 1:100.

For ICC, the sections were boiled in citrate buffer at pH 6 (20 minutes) to retrieve protein antigenicity. Endogenous peroxidases were blocked by incubation in 10% methanol 1% H_2_O_2_ solution (15 minutes), followed by 3% normal horse serum solution for 30 minutes. Afterward, the sections were incubated at 4°C overnight with one of the primary antibodies and were subsequently washed in PBS. After secondary antibody (biotinylated anti–mouse and –rabbit IgG; Abcam, ab64257) (30 minutes) incubation followed by streptavidin (Abcam, ab64269) (30 minutes) incubation, the peroxidase reaction was visualized with DAB and H_2_O_2_. Sections were slightly counterstained with hematoxylin. In addition, double-staining was performed to detect GFAP with IL-6, repeating the same process using IL-6 antibody in the same sections previously incubated with GFAP but revealing using DAB-Niquel (NH_4_NiSO_4_) solution. In this case, the immunoreaction resulted in a blue-gray precipitate; for this reason, sections were not counterstained with hematoxylin. Control of the immunostaining included omission of the primary antibody; no signal was obtained following incubation with only the secondary antibody.

For IF, the sections were boiled in citrate buffer at pH 6 (20 minutes) to retrieve protein antigenicity and were preincubated with a saturated solution of Sudan Black to block autofluorescence. Afterward, the sections were incubated at 4°C overnight with one of the primary antibodies and were subsequently washed in PBS. For IF, the sections were incubated with Alexa Fluor 488 (1:400, Molecular Probes) fluorescence secondary antibodies. Nuclei were stained with DRAQ5 (1:2,000, Biostatus). After washing, the sections were mounted in Immuno-Fluor mounting medium (ICN Biomedicals), sealed, and dried overnight. Sections were examined with a Leica TCS-SL confocal microscope.

### Statistics

Box-and-whisker plots show median, 25th and 75th percentiles, minimum, and maximum values and were analyzed using GraphPad Prism 7.0 (Mac OS X). Statistical significance was assessed with a 2-tailed unpaired *t* test or Mann-Whitney *U* test for 2 experimental groups. For experiments with 3 or more groups, 1-way ANOVA with Bonferroni’s multiple-comparison test as post hoc was used. Results were considered significant when *P* < 0.05.

### Study approval

Postmortem brain samples were obtained from the Institute of Neuropathology Brain Bank (a branch of the HUB-ICO-IDIBELL Biobank, Barcelona) following the Spanish guidelines of the Real Decreto 1716/2011 and the approval of the ethics committee. The generation and use of human iPSCs in this work were approved by the Spanish competent authorities (Commission on Guarantees concerning the Donation and Use of Human Tissues and Cells of the Carlos III National Institute of Health). All procedures adhered to internal and EU guidelines for research involving derivation of pluripotent cell line. All patients gave informed consent for the study using forms approved by the ethical Committee on the Use of Human Subjects in Research at Hospital Clinic in Barcelona.

### Data availability

Values for all data points found in graphs are in the Supporting Data Values file. All the sequencing data have been deposited at National Center for Biotechnology Information and are accessible through GEO Series accession no. GSE207713.

## Author contributions

MPE, AR, and AC conceived and designed the experiments; MPE, LBA, IFC, PAB, ADD, YRP, VB, and LM performed the experiments and acquired the data; JLM contributed analytic tools and analysed the data; LE, ET, AG, MJE MJO, IF, AR, and AC provided resources; MPE and AC wrote the paper; ET, IF, and AR reviewed the manuscript; and ADD edited the paper. All authors agreed to the order of authorship.

## Supplementary Material

Supplemental data

Supplemental table 2

Supporting data values

## Version 1 02/08/2024

Electronic publication
