# Supplementary material for: Blocking IL-6 signaling prevents astrocyte-induced neurodegeneration in an iPSC-based model of Parkinson’s disease
Source: JCI Insight. 2024 Feb 8;9(3):e163359. doi: 10.1172/jci.insight.163359 (PMC10967472; doi:10.1172/jci.insight.163359)
Supplement: Supplemental data [file jciinsight-9-163359-s243.pdf]

SUPPLEMENTAL INFORMATION

Blocking IL6 signaling prevents astrocyte-induced neurodegeneration in an iPSC-based model of Parkinson's disease

Meritxell Pons-Espinal[1,2]*, Lucas Blasco-Agell[1,2], Irene Fernandez-Carasa[1,2], Pol Andrés-Benito[1,3,4], Angelique di Domenico[1,2+], Yvonne Richaud[5,6], Valentina Baruffi[1,2], Laura Marruecos[7], Lluís Espinosa[7], Alicia Garrido[8,9], Eduardo Tolosa[8,9], Michael J. Edel[10,11], Manel Juan Otero[12], José Luis Mosquera[13], Isidre Ferrer[1,3,4], Angel Raya[5,6,14] and Antonella Consiglio[1,2,15] *

1. Department of Pathology and Experimental Therapeutics, Bellvitge University Hospital-IDIBELL, 08908 Hospitalet de Llobregat, Spain.
2. Institute of Biomedicine (IBUB) of the University of Barcelona (UB), 08028 Barcelona, Spain.
3. Neuropathology, Bellvitge Biomedical Research Institute (IDIBELL), Hospital Duran i Reynals, Hospitalet de Llobregat 08908, Spain.
4. Network Research Center of Neurodegenerative Diseases (CIBERNED), Hospitalet de Llobregat 08908, Spain
5. Institute for Neurodegenerative Diseases, University California San Francisco, San Francisco CA 94158, USA.
6. Regenerative Medicine Program, Bellvitge Biomedical Research Institute (IDIBELL), and Program for Clinical Translation of Regenerative Medicine in Catalonia (P-CMRC), Hospital Duran i Reynals, Hospitalet de Llobregat, 08908 Barcelona, Spain.
7. Centre for Networked Biomedical Research on Bioengineering, Biomaterials and Nanomedicine (CIBER-BBN), 28029 Madrid, Spain.
8. Institut Municipal d'Investigacions Mèdiques, Institut Hospital del Mar d'Investigacions Mèdiques, Parc de Recerca Biomèdica de Barcelona, Barcelona 08003, Spain.
9. Centre for Networked Biomedical Research on Neurodegenerative Diseases (CIBERNED), Madrid 28049, Spain.
10. Department of Neurology, Hospital Clínic de Barcelona, Institut d'Investigacions Biomèdiques August Pi i Sunyer (IDIBAPS), University of Barcelona (UB), 08036 Barcelona, Spain.
11. Autonomous University of Barcelona, Faculty of Medicine, Unit of Anatomy and Embryology, Barcelona, Spain.
12. 2. University of Western Australia, Faculty of Medicine, Discipline of Medical Sciences and Genetics, School of Biomedical Sciences, Perth 6009, Australia
13. Immunology Department–CDB, Hospital Clinic de Barcelona, Institut d'Investigacions Biomèdiques August Pi i Sunyer (IDIBAPS), University of Barcelona (UB), Barcelona 08036, Spain.
14. Bioinformatics Unit, Institut d'Investigació Biomèdica de Bellvitge – IDIBELL, L'Hospitalet de Llobregat, Spain.
15. Institució Catalana de Recerca i Estudis Avançats (ICREA), 08010 Barcelona, Spain
16. Department of Molecular and Translational Medicine, University of Brescia, Piazza del Mercato, 15, 25121 Brescia BS, Italy.

+ Current Address: Institute for Neurodegenerative Diseases, University California San Francisco, San Francisco CA 94158, USA

§ Correspondence and requests for materials should be addressed to: M.P.E. (email: meritxell.pons@ub.edu) or A.C. (email: consiglio@ub.edu)

Supplemental Information contains:

Supplementary Figures:

Figure S1. RNA-seq analysis between L2-PD and CTL astrocytes.

Figure S2. L2-PD astrocytes-related reactivity depends on increased L2 kinase activity.

Figure S3. IL6/IL6R signaling is involved in DA neuronal degeneration.

Figure S4. iPSC-derived astrocyte characterization from idiopathic PD patients.

Supplementary Table:

Table S1. List of iPSC lines used antibodies used in our studies.

Table S2. Results from GSEA analysis.

Table S3. List of primers used qRT-PCR used in our studies.

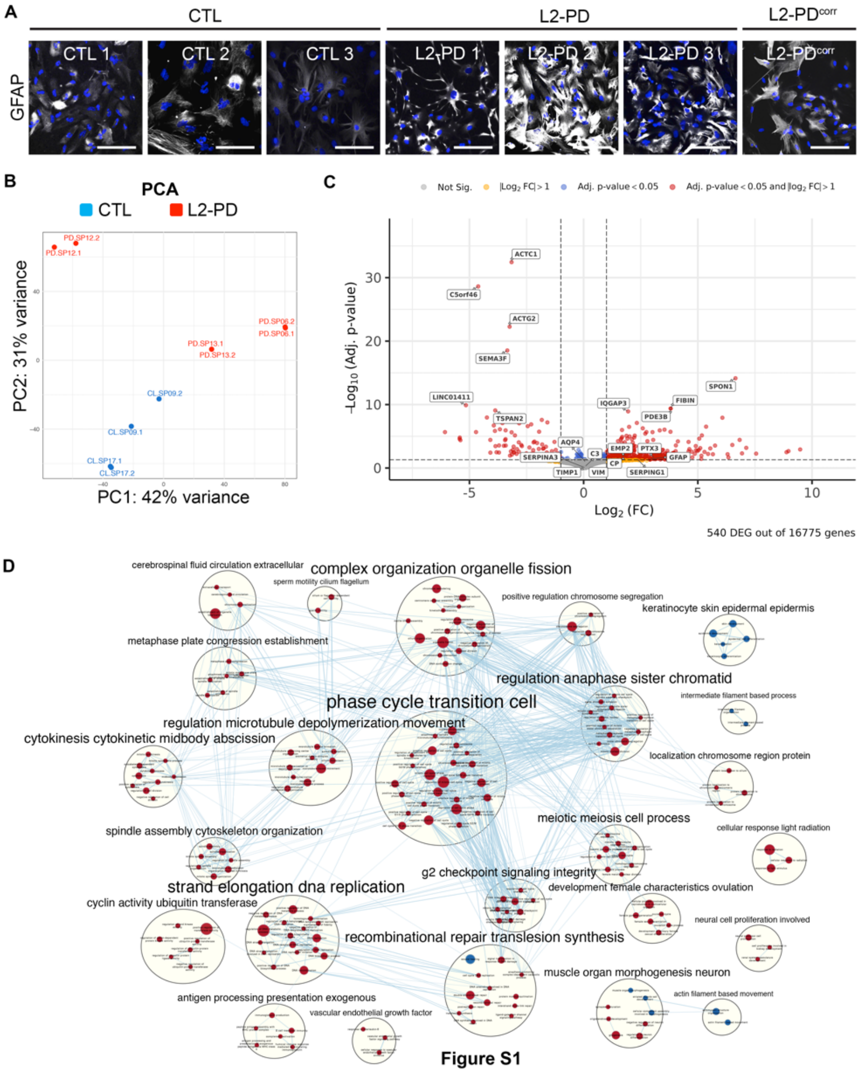

Figure S1

Supplemental Figure 1. RNA-seq analysis between L2-PD and CTL astrocytes. (**A**) Representative ICC images of astrocytes from three CTL iPSC lines (CTL 1: SP09, CTL 2: SP17, CTL 3: SP11 FLAG), three L2-PD iPSC lines (L2-PD 1: SP06, L2-PD 2: SP12 and L2-PD 3: SP13) and one isogenic iPSC line (L2-PD^{corr}: SP13wt/wt) staining positive for DAPI (blue) and GFAP (white) after 2 weeks in culture. Scale bar: 100 µm. (**B**) Representation of the first two dimensions from the Principal Component Analysis (PCA) showing the clustering patterns of CTL iPSC lines by the second component (blue dots; CTL 1: SP09 and CTL 2: SP17) and three L2-PD iPSC lines (red dots; L2-PD 1: SP06, L2-PD 2: SP12 and L2-PD 3: SP13). Both dimensions describe almost 72% of the whole variability. (**C**) Volcano plot showing expression changes of genes between L2-PD iPSC-derived astrocytes and Controls. Applied cutoffs are 0.05 for the adjusted p-values and 1 for the log(Fold Change). Red dots represent up and down regulated genes. Blue dots indicate statistically significant genes with an absolute value of log2(Fold Change) equal or lower than 1. Orange dots are genes showing high levels of log2 (Fold Change) but they are not statistically significant. Labeled genes are the top 10 differentially expressed genes and those genes that have been validated by qPCR. (**D**) Annotated enrichment map build with the Enrichment Cytoscape App and AutoAnnotate Cytoscape App. The GO BP gene sets are those that showed a False Discovery Rate lower than 0.1. Clusters of gene sets were identified with the mclust method based on a GO semantic similarity calculated with the Jiang method approach.

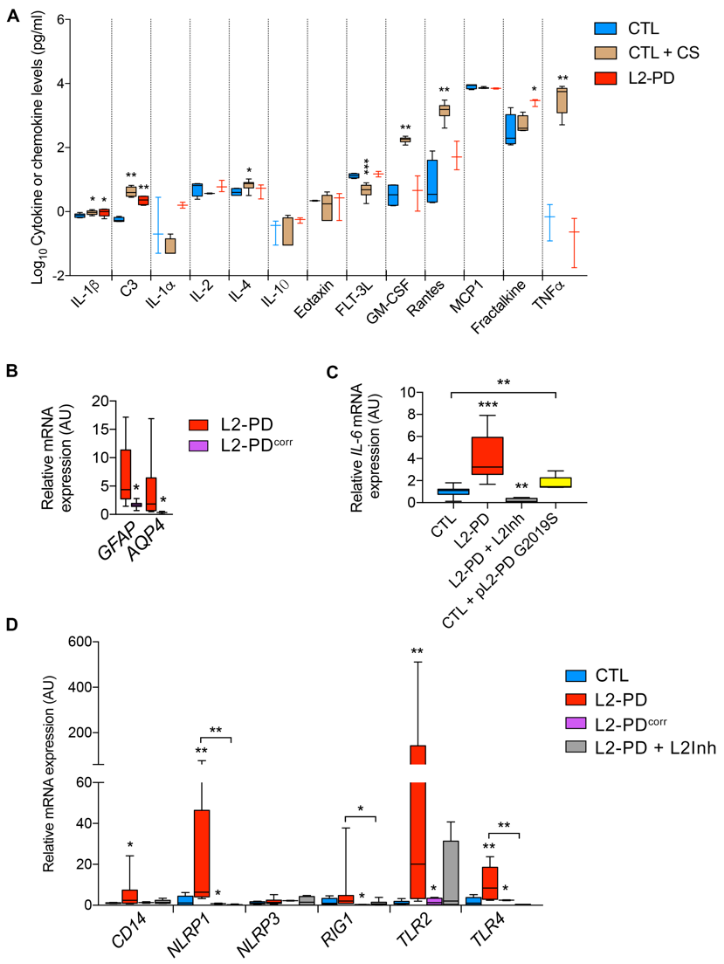

Figure S2

Supplemental Figure 2. L2-PD astrocytes-related reactivity depends on increased L2 kinase activity. (**A**) Cytokine and chemokine levels released by CTL and L2-PD astrocyte conditioned medium (ACM) after 2 weeks of cell culture. CTL astrocytes were treated for 48h with C1q, TNFa and IL1a as positive control. (**B**) Relative mRNA expression of *GFAP* and *AQP4* in L2-PD and L2-PD^{corr}. (**C**) Relative *IL6* mRNA expression in CTL, L2-PD, L2-PD-treated astrocytes with LRRK2-kinase inhibitor (1uM) or CTL astrocytes after 48h transfection with a plasmid pLRRK2-G2019S to overexpress LRRK2^{G2019S}. (**D**) Gene expression analysis using qRT-PCR showing several inflamed-dependent receptors in CTL, L2-PD, L2-PD^{corr} or L2-PD-treated astrocytes with LRRK2-kinase inhibitor (1uM). Box-and-whisker plots show median, 25th and 75th percentiles, minimum, and maximum values (n=3 experiments). One-way ANOVA Bonferroni as post-hoc. Student t-test or Mann-Whitney test for non-parametric conditions were used when only two groups were compared. *p<0.05; **p<0.01; ***p<0.001.

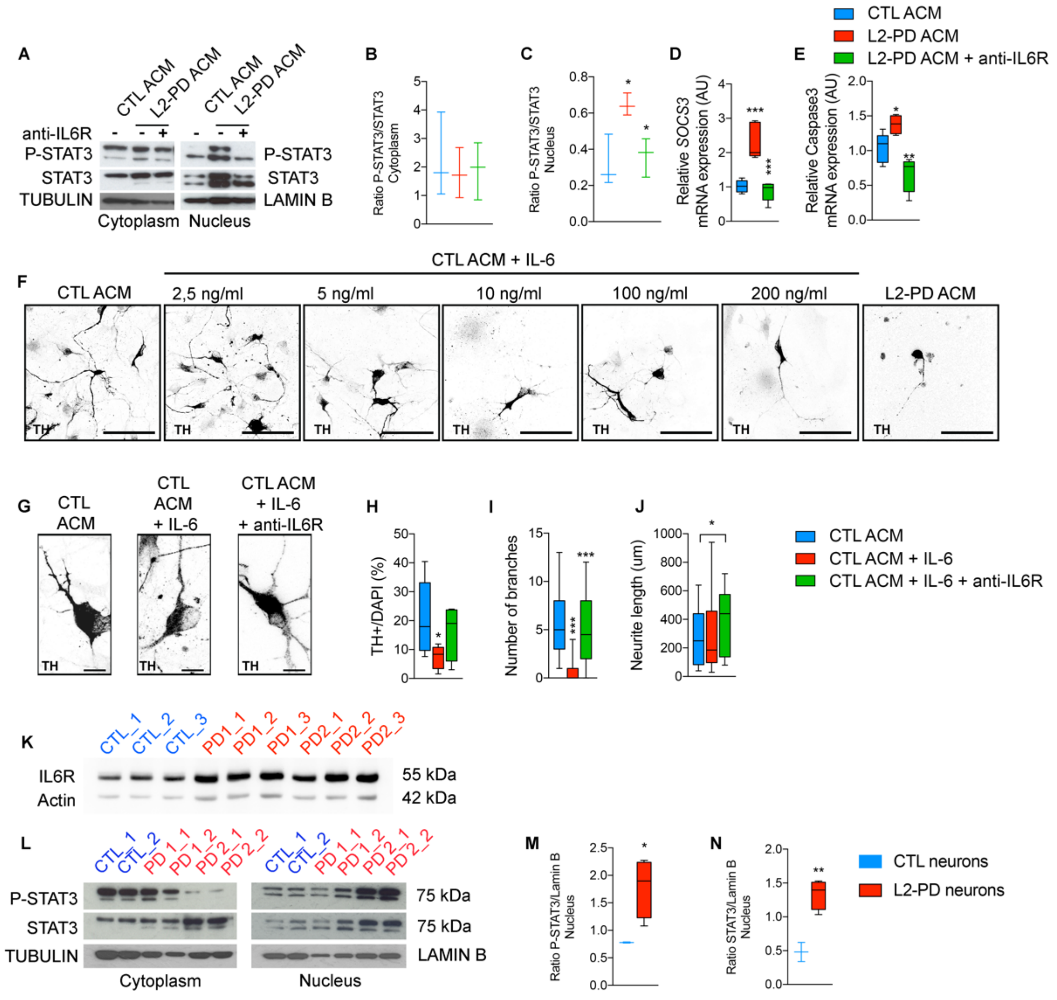

Figure S3

Supplemental Figure 3. IL6/IL6R signaling is involved in DA neuronal degeneration. (**A**) Western blot for STAT3 and p-STAT3 in the cytoplasm and nuclear fractions of iPSC-derived CTL neurons treated with CTL ACM or L2-PD ACM with anti-IL6R antibody Tocilizumab. (**B-C**) Quantification of protein p-STAT3/STAT ratio in the cytoplasm (**B**) or nucleus (**C**). (**D**) Relative *SOCS3* mRNA levels. (**E**) Relative *Caspase 3* mRNA levels. (**F**) Representative ICC images of DAn (TH, black) from CTL SP11 neurons treated with either CTL ACM alone or with increasing IL6 concentrations (2.5 ng/ml to 200ng/ml), or with L2-PD ACM during one week. (**G**) Representative ICC images of tyrosine hydroxylase (TH, black) from CTL SP11 neurons with CTL ACM treated with IL6 (10ng/ml) and anti-IL6R Tocilizumab (10ug/ml) after one week in culture. Scale bar: 20 µm. (**H**) Percentage of TH+ cells respect to DAPI in CTL SP11 neurons when cultured with ACM plus IL6 and anti-IL6R antibody. (**I**) Number of branches and (**J**) neurite length of CTL SP11 TH+ neurons. Box-and-whisker plots show median, 25th and 75th percentiles, minimum, and maximum values (n=3 experiments; 30 neurons per experiment per condition). (**K**) Western blot for IL6R of iPSC-derived CTL neurons (SP11) and L2-PD neurons (SP12 and SP13). (**L**) Western blot for STAT3 and p-STAT3 in the cytoplasm and nuclear fractions of iPSC-derived CTL neurons (SP11) and L2-PD neurons (SP06 and SP13) treated with L2-PD ACM. (**M**) Quantification of protein p-STAT3/ Lamin B ratio in the nucleus. (**N**) Quantification of protein STAT3/ Lamin B ratio in the nucleus. Box-and-whisker plots show median, 25th and 75th percentiles, minimum, and maximum values (n=3 experiments). One-way ANOVA Bonferroni as post-hoc. Student t-test or Mann-Whitney test for non-parametric conditions were used when only two groups were compared. *p<0.05; **p<0.01; ***p<0.001.

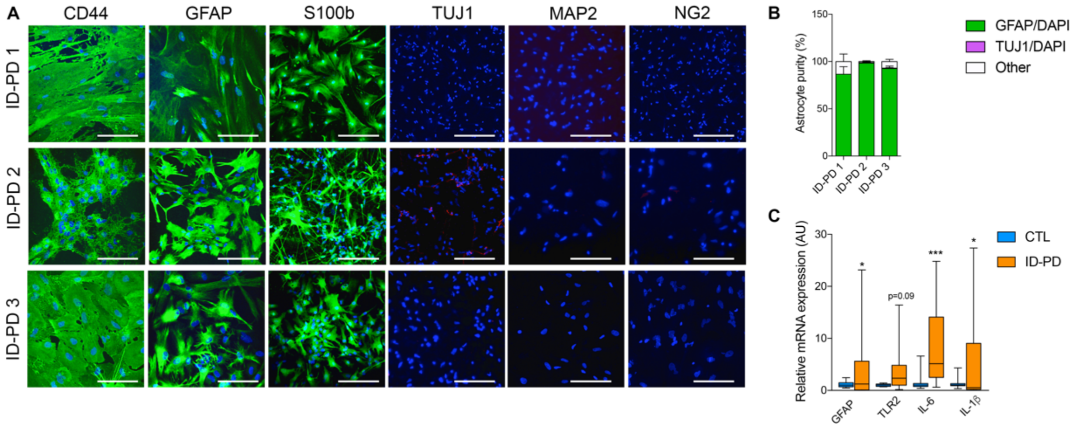

Figure S4

Supplemental Figure 4. iPSC-derived astrocyte characterization from idiopathic PD patients. (**A**) Representative ICC images of astrocytes derived from idiopathic PD iPSC lines (ID-PD 1: SP04, ID-PD 2: SP08 and ID-PD 3: SP16) staining positive for CD44 (astrocytic precursor marker), GFAP (general astrocytes), S100b (mature astrocytes), and negative for TUJ1 (immature neurons), MAP2 (mature neurons), and NG2 (oligodendrocytes) expression. Number of independent experiments per astrocyte line generated = 3. Scale bar, 100 µm. (**B**) Astrocyte cultures are composed of approximately 95% astrocytes, 4% neurons, and 1% other. (**C**) Relative *GFAP*, *TLR2*, *IL6* and IL*1b* mRNA expression analysis using qRT-PCR. Box-and-whisker plots show median, 25th and 75th percentiles, minimum, and maximum values (n=3 experiments; Form factor, Mean GFAP and C3 intensity was performed from 30 astrocytes per experiment per condition). Student t-test or Mann-Whitney test for non-parametric conditions were used when only two groups were compared. *$p<0.05$; **$p<0.01$; ***$p<0.001$.

Supplemental Table 1. Summary of iPSC lines used

Table S1. Summary of iPSC Lines Used

Patient			Disease			
**Code**	**Status**	**Sex**	**Age at Biopsy**	**Age of Onset**	**Family history**	**Mutation**
SP09	Control	M	66	N/A	No	No
SP17	Control	M	52	N/A	No	No
SP11-FLAG	Control	F	48	N/A	No	No
SP11	Control	F	48	N/A	No	No
SP13wtwt	PD LRRK2 Mutant Corrected	F	68	57	Yes	No
SP06	PD LRRK2 Mutant	M	44	33	Yes	LRRK2G2019S
SP12	PD LRRK2 Mutant	F	63	49	Yes	LRRK2G2019S
SP13	PD LRRK2 Mutant	F	68	57	Yes	LRRK2G2019S
SP04	Idiopathic PD	M	46	40	No	No
SP08	Idiopathic PD	F	66	60	No	No
SP16	Idiopathic PD	F	51	48	No	No

Supplemental Table 2. GSEA analysis results

- Differential Expression Analysis (DEA):

- DEA results (PD vs Control): Table of differential expression results derived from the analysis conducted with the Bioconductor DESeq2 package when comparing PD vs Control = PD – Control.

- Num. DEG: Summary table providing the number of differentially expressed genes according with different selection criteria.

- Gene Set Enrichment Analysis (GSEA):

- GSEA pre-ranked genes: Table of pre-ranked genes considered for the GSEA.

- GSEA results: Table of enrichment results using the GO biological processes (BP) derived from the collection C5 provided in MSigDB and analyzed with the GSEA function from the Bioconductor packages clusterProfiler.

- Post-Processing Enrichment Analysis: Simplification of GO Terms Redundancy:

- Simplified GSEA results: Table with the clusters assigned to the significant GO BP gene sets (p.adjust<0.1) applying the mclust method for the three different GO semantic similarity matrices.

- Semantic similarity (Relevance): Matrix containing the similarity of GO terms estimated with the function GO_similarity from the Bioconductor package simplifyEnrichment, using the Relevance method.

- **Semantic similarity (Jiang):** Matrix containing the similarity of GO terms estimated with the function GO_similarity from the Bioconductor package simplifyEnrichment, using the Jiang method.

- Semantic similarity (Wang): Matrix containing the similarity of GO terms estimated with the function GO_similarity from the Bioconductor package simplifyEnrichment, using the Wang method.

- Ann. enrich. map (selected clusters): Table with the genes corresponding to the clusters of GO BP gene sets of interest.

Supplemental Table 3. Primers used for qRT-PCR.

Primer ID	Forward (5' – 3')	Revers (5' – 3')
GFAP	CCTCTCCCTGGCTCGAATG	GGAAGCGAACCTTCTCGATGTA
AQP4	GGTAAGTGTGGACCTTTGTGT	CAAAGCAAAGGGAGATGAGAAC
VIMENTIN	GCCCTAGACGAACTGGGTC	GGCTGCAACTGCCTAATGAG
ACTIN	AGGCCAACCGCGAGAAG	ACAGCCTGGATAGCAACGTACA
TLR2	ATCCTCCAATCAGGCTTCTCT	ACACCTCTGTAGGTCACTGTTG
TLR4	ATATTGACAGGAAACCCCATCCA	AGAGAGATTGAGTAGGGGCATTT
CD14	ACTTGCACTTTCCAGCTTGC	GCCCAGTCCAGGATTGTCAG
NLRP1	GGACTGACGATGACTTCTGG	ATCACAAAGCAGAGACCCG
NLRP3	GGAGAGACCTTTATGAGAAAGCAA	GCTGTCTTCCTGGCATATCACA
RIG1	GCAGGATTTGTAAAGCCCTGTT	CACTGATAATGAGGGCATCATTATATTT
IL6	AATTCGGTACATCCTCGACGG	GGTTGTTTTCTGCCAGTGCC
IL6R	CCCATCCCTGACGACAA	ACTGCTAACTGGCAGGAGAA
IL1b	GCTGAGGAAGATGCTGGTTC	TCCATATCCTGTCCCTGGAG
C3	AAAAGGGGCGCAACAAGTTC	GATGCCTTCCGGGTTCTCAA
SOCS3	AGCAGCGATGGAATTACCTGGAAC	TCCAGCCCAATACCTGACACAGAA
CASPASE3	CAAACTTTTTCAGAGGGGATCG	GCATACTGTTTCAGCATGGCAC
LCN	CCCAGCCCCACCTCTGA	CTTCCCCTGGAATTGGTTGTC
TIMP1	TTGTGGGACCTGTGGAAGTA	CTGTTGTTGCTGTGGCTGAT
SERPIN3N	AGCAGTGGGGCTCTCAGTAA	ATAAGCAGACAGGGCCACAC
PTX3	CATCCAGTGAGACCAATGAG	GTAGCCGCCAGTTCACCATT
CP	ACGGCCATAGCTTCCAATACAA	AGTTGTATGCTTCCAGTCTTCT
SERPING1	AACCTGTGGCCCATTTCATT	TCTGGGGTACCAGGATCAC
OSMR	ACCTGCCACAGAGTACATGG	GCTCCAAGCTCACAATTCTCCA
EMP2	AGGGAATACATGGTTTACTCCA	AGAGAGATTGGCCAGCAAAA
